# Elucidating the ecophysiology of soybean pod-sucking stinkbug *Riptortus pedestris* (Hemiptera: *Alydidae*) based on de novo genome assembly and transcriptome analysis

**DOI:** 10.1186/s12864-024-10232-2

**Published:** 2024-04-02

**Authors:** Chade Li, Wenyan Nong, Delbert Almerick T. Boncan, Wai Lok So, Ho Yin Yip, Thomas Swale, Qi Jia, Ignacio G. Vicentin, Gyuhwa Chung, William G. Bendena, Jacky C. K. Ngo, Ting Fung Chan, Hon-Ming Lam, Jerome H. L. Hui

**Affiliations:** 1 Center for Soybean Research of the State Key Laboratory of Agrobiotechnology, The Chinese University of Hong Kong, Shatin, HKSAR China; 2 Simon F.S. Li Marine Science Laboratory, School of Life Sciences, The Chinese University of Hong Kong, Shat-in, HKSAR China; 3 Institute of Environment, Institute of Energy and Sustainability, The Chinese University of Hong Kong, Shatin, HKSAR China; 4 Dovetail Genomics, Scotts Valley, CA USA; 5 Key Laboratory for Genetics Breeding and Multiple Utilization of Crops, Ministry of Education/College of Crop Science, Fujian Agriculture and Forestry University, Fuzhou, 350002 PR China; 6 Instituto Nacional de Tecnologia Agropecuaria, Avenida Rivadavia, Ciudad de Buenos, 1439 Argentina; 7 Department of Biotechnology, Chonnam National University, Yeosu, 59626 Korea; 8 Department of Biology, Queen’s University, 116 Barrie St, Kingston, ON K7L 3N6 Canada

**Keywords:** Soybean, Stinkbug, MicroRNA, Sesquiterpenoid, Ecdysteroid, Neuropeptide, Transcriptome

## Abstract

Food security is important for the ever-growing global population. Soybean, *Glycine max* (L.) Merr., is cultivated worldwide providing a key source of food, protein and oil. Hence, it is imperative to maintain or to increase its yield under different conditions including challenges caused by abiotic and biotic stresses. In recent years, the soybean pod-sucking stinkbug *Riptortus pedestris* has emerged as an important agricultural insect pest in East, South and Southeast Asia. Here, we present a genomics resource for *R. pedestris* including its genome assembly, messenger RNA (mRNA) and microRNA (miRNA) transcriptomes at different developmental stages and from different organs. As insect hormone biosynthesis genes (genes involved in metamorphosis) and their regulators such as miRNAs are potential targets for pest control, we analyzed the sesquiterpenoid (juvenile) and ecdysteroid (molting) hormone biosynthesis pathway genes including their miRNAs and relevant neuropeptides. Temporal gene expression changes of these insect hormone biosynthesis pathways were observed at different developmental stages. Similarly, a diet-specific response in gene expression was also observed in both head and salivary glands. Furthermore, we observed that microRNAs (bantam, miR-14, miR-316, and miR-263) of *R. pedestris* fed with different types of soybeans were differentially expressed in the salivary glands indicating a diet-specific response. Interestingly, the opposite arms of miR-281 (-5p and -3p), a miRNA involved in regulating development, were predicted to target *Hmgs* genes of *R. pedestris* and soybean, respectively. These observations among others highlight stinkbug’s responses as a function of its interaction with soybean. In brief, the results of this study not only present salient findings that could be of potential use in pest management and mitigation but also provide an invaluable resource for *R. pedestris* as an insect model to facilitate studies on plant-pest interactions.

**Supplementary Information:**

The online version contains supplementary material available at 10.1186/s12864-024-10232-2.

## Introduction

Soybean (*Glycine max* (L.) Merr.) is one of the most important agricultural crops in the world, and addressing current and emerging biotic challenges is necessary to safeguard both quality and yield. Like other crops, soybeans are vulnerable to damage caused by different plant pests. Microbial pathogens such as viruses, bacteria, fungi and protozoa cause diseases, while insect infestations (or incursions) cause damage and vector diseases. Most soybean insect pests are classified under the Orders Lepidoptera, Hemiptera and Coleoptera, and those that feed on pods are recognized as key pests capable of causing significant reduction in yield and seed quality [1, 2]. Hence, understanding the biology and ecology of these pests is imperative to develop efficient management and mitigation strategies. While studies have focused on insect development and bacterial endosymbiosis of insect pests, plant–insect interactions constitute an important yet often neglected aspect. For instance, host-plant resistance (HPR) leverages endogenous plant defenses for improved and sustainable crop production [3, 4]. Hemipterans are a common target of HPR, but no other group is more targeted than the aphids [5, 6]. However, the emergence of resistance-breaking aphid biotypes in different plant-aphid systems including soybeans has prevented the full utilization of HPR [7, 8]. The stem-borer soybean aphid (*Aphis glycines*) – as an example – is an important pest in East Asia and in North America [9]. Its genome has been sequenced and assembled providing a valuable genomics resource. Notwithstanding that *A. glycines* has evolved resistance-breaking (virulent) biotypes, *A. glycines* is poised as an insect model to study soybean-pest interactions [10–12].

Meanwhile, the soybean pod-sucking stinkbug *Riptortus pedestris* is an emerging and a major insect pest of soybean in Asia that cause damage to crop by piercing and sucking the pods, seeds, stems and leaves [13]. Multiple species of stinkbugs were reported to have resulted in soybean yield loss in the US more than any other invertebrate pests in 2021 (doi.org/10.31274/cpn-20230511-0), while there has been an increasing number of reports of *R. pedestris* emergence in East, South and Southeast Asia [14–17]. Interestingly, *R. pedestris* has a foraging preference for leguminous crops from which it derives nutritional requirements for reproduction and development [18]. This insect feeds through mouthparts that form a stylet sheath allowing saliva with digestive enzymes to breakdown tissue and to facilitate sucking of plant fluids. Stinkbug-damaged soybeans eventually result in reduced seed nutritional quality and yield, and the number of seeds successfully germinating is also drastically affected [1, 14, 15, 19]. Furthermore, the soybean staygreen syndrome has been demonstrated to be more piercing and sucking (*R. pedestris*) related than what was initially thought to be pathogen related [14, 20]. Typically, plants respond by emitting substances for defense [21], and whether or how this occurs (mechanistic insights) in soybean-pest interaction is a relevant topic that warrants investigation. There have been no reports of HPR in soybeans against stinkbugs. Unlike typical hemipterans that can be eliminated with insecticides, *R. pedestris* has a hefty flying ability to escape and to reinvade crop fields during and after insecticide application. Hence, pest control strategies for *R. pedestrians* remain ineffective due to its evasiveness and the emergence of insecticide-resistant populations among others [22, 23].

Effectors are small proteins secreted into the insect saliva during feeding. These effectors work for or against the insect by suppressing or activating plant defenses [24–26]. Investigating this aspect may provide a better understanding of soybean-pest interaction, and ways of utilizing this knowledge for HPR offers a potential and an appealing target for mitigating *R. pedestris* infestation [17, 25, 27–31]. Interestingly, secreted proteins from *R. pedestris* that instigate plant cell death have been identified [32, 33], and cross-kingdom species interactions through plant–insect microRNAs have also been reported [34]. However, how interaction occurs between soybeans and stinkbugs remains unknown, and profiling the gene expression changes as a result of this interaction is thus necessary. Uncovering the interaction of *R. pedestris* and soybean through mRNA and miRNA (transcriptome) from a global and/or tissue-specific perspective is expected to provide insights on *R. pedestris* ecophysiology.

Thus, studying the interaction of soybean and its insect pest *R. pedestris* is of practical and scientific importance. In this study, we present a high-quality genome assembly of *R. pedestris*, its messenger RNA (mRNA) and microRNA (miRNA) transcriptomes representing different developmental stages and organs (head and salivary glands), and the miRNA profile of the salivary glands and the head subjected on different soybean diets.

## Materials and methods

### Insect culture

**Fig. 1:**
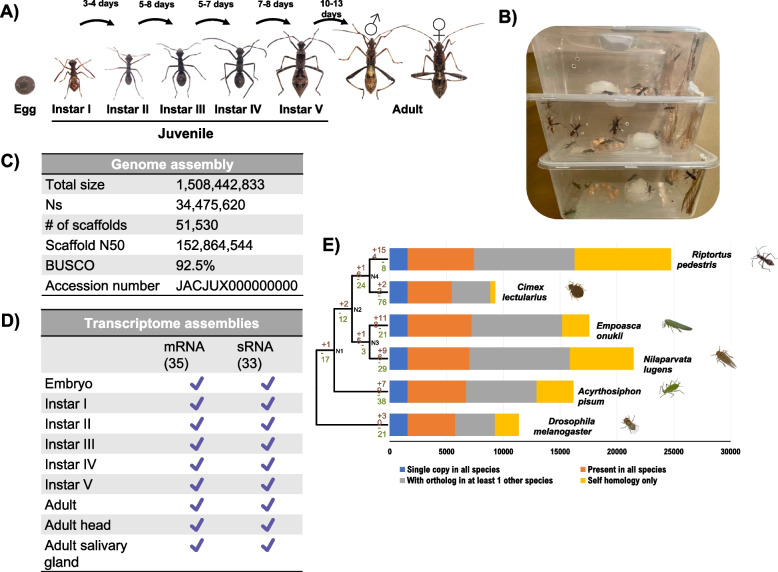
**A** Schematic diagram showing the life cycle of stinkbug *Riptortus pedestris*. **B** Laboratory/culture setup of *R. pedestris* culture in the laboratory. **C** Genome assembly statistics. **D** Summary of transcriptome sequencing (mRNA and sRNA). **E** Phylogenetic tree based on single-copy orthologs among hemipteran insects with *D. melanogaster* as outgroup

*R. pedestris* were captured by luring insects in pheromone traps on campus grounds of the Chinese University of Hong Kong, and the resulting insect cultures were maintained under laboratory conditions as previously described by [22] with modifications. *Riptortus pedestris* were reared in HDPE (plastic) containers with hanging PP (plastic) twine (liners) for oviposition, and they were provided with soybean seeds and distilled water supplemented with 0.05% L-ascorbic acid under 14 h/10 h light/dark light cycle at 25℃ and 74% relative humidity (RH) (Fig. 1B). The species was identified and authenticated based (a) on the existence of white spots along the lateral thorax (Supplementary Fig. 1) and (b) with mitochondrial cytochrome oxidase subunit I (COI) gene as barcoding/taxonomic marker.

### Genome and transcriptome sequencing across developmental stages

Genomic DNA (gDNA) was extracted from a single adult individual with E.Z.N.A.® Insect DNA Kit (Omega Bio-tek). gDNA was outsourced to Novogene (HK) Company Limited (Novogene) for 10X linked-read sequencing. At the beginning of the experiment, the gender of the insect used was undetermined and was subsequently validated to be a female based on XO sex determination system by aligning 10X linked-reads to the genome assembly of [35] including their male (SRR12977074) and female (SRR12897696) sequencing reads as benchmarks (Supplementary Table 15). Meanwhile, additional adult insects were outsourced to Dovetail Genomics for Hi-C sequencing. In addition, gDNA was also subjected to Nanopore sequencing and Arima Hi-C sequencing for scaffolding. Meanwhile, total RNA from *R. pedestris* representing different developmental stages including the embryo, instars (I to V) and adult were extracted with TRIzol™ Reagent (ThermoFisher Cat. No. 15596026) and outsourced to Novogene for messenger RNA (mRNA) and small/microRNA RNA (sRNA/miRNA) sequencing. Instars were staged based on the number of molts (i.e., counting the exuviae) each had in isolated containers.

### Genome assembly

Chromium whole-genome sequencing reads were initially used to generate a de novo assembly with Supernova v2.1.1 with default parameters [36], https://support.10xgenomics.com/de-novo-assembly/software/pipelines/latest/using/running). Then, dedupe contigs of the pseudohap style output, shotgun reads and Dovetail Chicago sequencing reads were fed to HiRise (January 2020 release) – a tool designed for handling proximity ligation data to scaffold genome assemblies [37]. Following an iterative approach, shotgun and Dovetail Chicago sequencing reads were aligned to the draft sequence assembly using a modified SNAP (20,131,129 release) read mapper (http://snap.cs.berkeley.edu). The physical separation of Dovetail Chicago sequencing read pairs mapped within draft scaffolds were analyzed by HiRise to generate a likelihood model of genomic distance between read pairs. The resulting model was used to identify and break putative misjoins, to score prospective joins and to make joins above a threshold. Meanwhile, Arima Hi-C paired-end reads were processed with Juicer (v1.5) and Juicer Tools (v1.14.08) following steps necessary to generate the required input files for 3D-DNA [38, 39]. Contiguous DNA resulting from Chromium and Chicago-HiRise were scaffolded with 3D-DNA (v180114,-m haploid -i 15,000 -g 500). Gap closing was performed with error-corrected Nanopore reads using TGS-GapCloser [31, 40]. Deduplication was carried out to remove duplicates, while scaffolds/contigs shorter than 10 kbp were excluded [41]. The mapping rates of the RNA-seq data (instars I-V and adult) were examined by aligning sequencing reads against the publicly available *R. pedestris* genome [35] and to *R. pedestris* genome assembly in this study. A comparative analysis was conducted relative to the genome generated by [35]. Here, relevant genome assembly statistics was generated. The completeness, synteny and repetitive elements were assessed with BUSCO v5.5.0 (Supplementary Table 16) [42], SyMAP v5.4.6 ( [43, 44] and Earl Grey v1.3 [45] (Supplementary Fig. 27 and Supplementary Tables 16 and 17), respectively.

### Gene model prediction

The gene models were trained and predicted with Funannotate (v1.8.4, https://github.com/nextgenusfs/funannotate) [46] with parameters “–repeats2evm –protein_evidence uniprot_sprot.fasta –genemark_mode ET –optimize_augustus –busco_db arthropoda –organism other –max_intronlen 350,000”. The gene models from several prediction sources, including GeneMark v3 [47], Augustus HiQ v3.4.0, PASA v2.5.3 [48], Augustus [49], GlimmerHMM v3.0.4 [50] and SNAP [51], were passed through EvidenceModeler v2.1.0 (EVM) [48] to generate the gene model annotation files. PASA was used to update EVM consensus predictions, add untranslated (UTR) annotations and generate models for alternatively spliced transcripts (isoforms). Subsequently, protein-coding gene models were analyzed against the National Center for Biotechnology Information (NCBI) nr and Swiss-Prot databases using DIAMOND-BLASTp v0.9.24 [52] with parameters "–more-sensitive –evalue 1e-3". Similarly, RNA-seq reads were mapped to protein-coding gene models using HISAT2 v2.1.0. Gene count matrix was generated with StringTie v2.1.1 [53] for gene expression analyses. Gene models without detected homology to known proteins and whose existence unsupported by RNA-seq data were excluded from the annotation. Finally, the prediction of repetitive elements was performed as described previously by [54]. Comparative genome analysis was performed among representative hemipteran species and *R. pedestris* including *Drosophila melanogaster* by aligning 1,577 single-copy orthologs (identified by OrthoFinder v2.5.4 [55] with MAFFT v7.520 [56] from which a maximum likelihood (ML) phylogenetic tree using IQ-TREE2 v2.2.0.3 [57] was constructed.

### Gene family analysis

Juvenile hormone (JH) and ecdysteroid hormone biosynthesis genes from both *Halyomorpha halys* and *Drosophila melanogaster* were retrieved from the Kyoto Encyclopedia of Genes and Genomes (KEGG) [58–60] database and FlyBase [61]. These sequences were used to identify orthologs in the genome using BLASTp v2.2.31 search with an E-value threshold of 10e-3. Putative orthologs were further analyzed by BLASTp search against NCBI nr database. The gene expression heatmap of the juvenile hormone and ecdysteroid hormone biosynthesis pathway genes were visualized with TBtools v1.132 [62]. Similarly, putative neuropeptide orthologs were confirmed by reciprocal BLASTp search (E-value = 10e-3) against NCBI nr database. Amino acid sequences of neuropeptide preprohormones were translated and aligned with MEGA v7.0 [63], while the signaling sequences were analyzed with SignalP v3.0 [64]. Manual inspection for potential mature peptide processing sites in the prepropeptide sequences were performed following the suggested guidelines of [65].

### Head and salivary gland transcriptome sequencing

*R. pedestris* male adults were starved for 2 days – precluding both soybeans and water – prior to feeding of different types of seeds (cultivated soybean (C08), wild soybean (W05) and common bean) for one week. In this experiment, all insects underwent fasting including those that were fed afterwards. *R. pedestris* male adults that did not receive food and water served as controls. The experiment was conducted in an incubator with 14 h/10 h light/dark light cycles at 25℃ and 70% RH. Prior to *R. pedestris* dissection, individuals were sterilized following the method of [66] with modifications. The surface of insects was sterilized with 70% ethanol, followed by rinsing with sterilized phosphate-buffered saline (PBS) buffer. Dissection was performed in a Petri dish with PBS buffer. Meanwhile, total RNA from the head and the salivary glands were extracted using TRIzol™ Reagent (ThermoFisher Cat. No. 15596026) and outsourced to Novogene for mRNA and sRNA sequencing. Three (3) individuals per control and feeding groups were used for head mRNA and sRNA sequencing. For salivary gland mRNA sequencing, two (2) individuals were used as controls, while three (3) individuals per feeding group were obtained from adults fed with C08, W05 and common bean. C08 and W05 represent cultivated and wild soybean species, respectively. Common bean was included to rule out the possibility that the observed soybean response was due (or partly due) to the act of feeding (confounding factor) after starvation. For salivary gland sRNA sequencing, two (2) individuals per control and feeding groups were used, while three (3) individuals were used for common bean feeding group (Supplementary Tables 2 and 3).

### miRNA annotation, quantification and target prediction

For *R. pedestris* miRNA annotation, quality-checked (trimmed and filtered) sRNA sequencing reads were mapped to the genome assembly and quantified using mapper.pl and quantifier.pl modules of miRDeep2 v2.0.0.8, respectively [67]. Predicted conserved miRNAs were manually checked by BLASTn search in miRBase v22.1 [68] with default parameters. Potential miRNAs were further aligned with reference miRNAs taken from MirGeneDB v2.1 [69]. The schematic diagram of miRNA distribution on scaffolds was visualized by TBtools [62]. Differential expression analysis (Supplementary Table 14) was conducted with Degust v4.0.0 [70] with parameters: CPM ≥ 1 in at least two (2) samples, FDR ≤ 0.05, and |log_2_FC|≥ 1. Conserved miRNAs which were expressed in all biological replicates in salivary glands were further processed for target prediction on i) soybean terpenoid pathway genes on SoyKB [71] with psRNATarget v16 (20,110,331 release) [72] on default parameters, and ii) on sesquiterpenoid pathway genes in *R. pedestris* with miRanda v3.3a with parameters “-strict -quiet” [73] and RNAhybrid v2.1.2 “-p 0.05 -e -10 -s 3utr_fly -f 2,7” [74]. For comparative analysis of the conserved hemipteran miRNAs, high quality genomes of hemipteran representative species were retrieved, and BLAST search was performed with the miRNA hairpin sequences on the databases with an E-value threshold of 10e-5. The resulting list of miRNAs were also compared against existing records in InsectBase v2.0 [75].

### RT-qPCR validation

cDNA was synthesized using iScript gDNA Clear cDNA Synthesis Kit (BIO-RAD). Quantitative reverse transcription PCR (RT-qPCR) was performed (1 cycle of 95 °C for 30 s, followed by 40 cycles of 95 °C for 5 s and 55 °C for 20 s) on three (3) head biological replicates with CFX96 Touch Real-Time PCR machine (BIO-RAD) and iTaq Universal SYBR® Green Supermix (BIO-RAD). *Tubulin* was selected as a housekeeping gene [76]. The amplification efficiency of primers was calculated by preparing serial dilutions of the target cDNA, while a linear regression curve (C_t_ values versus log [cDNA] dilutions) was fitted through the data points to calculate the slope of the line. Finally, efficiency (E) was calculated based on the following equation: $-1+{10}^{(-\frac{1}{slope})}$. For reference, the housekeeping gene *tub* and *jhamt* had amplification efficiencies of 106.12% and 100.58%, respectively. Oligonucleotide primers’ information is available in Supplementary Table 12.

## Results

### Riptortus pedestris’ life cycle – a resource for studying plant-pest interactions

The life cycle of *R. pedestris* consists of seven (7) distinct developmental stages characteristic of hemimetabolous insects: embryo/egg, first to fifth instar and adult. The number of days from first instar to adult ranged from 32 to 37 days. Adults emerged approximately one (1) month from first instar at 22–25℃ (Fig. 1A). In this study, a high-quality reference genome of *R. pedestris* with genome size around 1.5 Gbp was assembled with completeness of 92.5% based on BUSCO and scaffold N50 of 152 Mbp (Fig. 1C, Supplementary Tables). Meanwhile, a comparative analysis (Supplementary Tables 16 and 17, Supplementary Fig. 27) with the existing genome assembly of *Riptortus pedestris* by [35] demonstrated similar genome organization and repeat profile. RNA-seq representing the mRNA transcriptomes from different developmental stages were generated, annotated and analyzed, while RNA-seq and sRNA-seq (miRNA) of the head and salivary glands of the adults subjected to different soybean/bean diets were likewise generated and analyzed (Fig. 1D, Supplementary Tables 2 and 3). Based on single-copy orthologs, the ML tree’s topology shows the phylogenetic affiliation of *R. pedestris* with respect to other hemipteran species and to *Drosophila melanogaster* where *Cimex lectularius* as the more closely related hemipteran (Fig. 1E). Collectively, this study provides useful resources for conducting functional genomics and validation studies among others.

### Insect hormone biosynthesis: Sesquiterpenoid (Juvenile) and Ecdysteroid (Molting) hormones

**Fig. 2:**
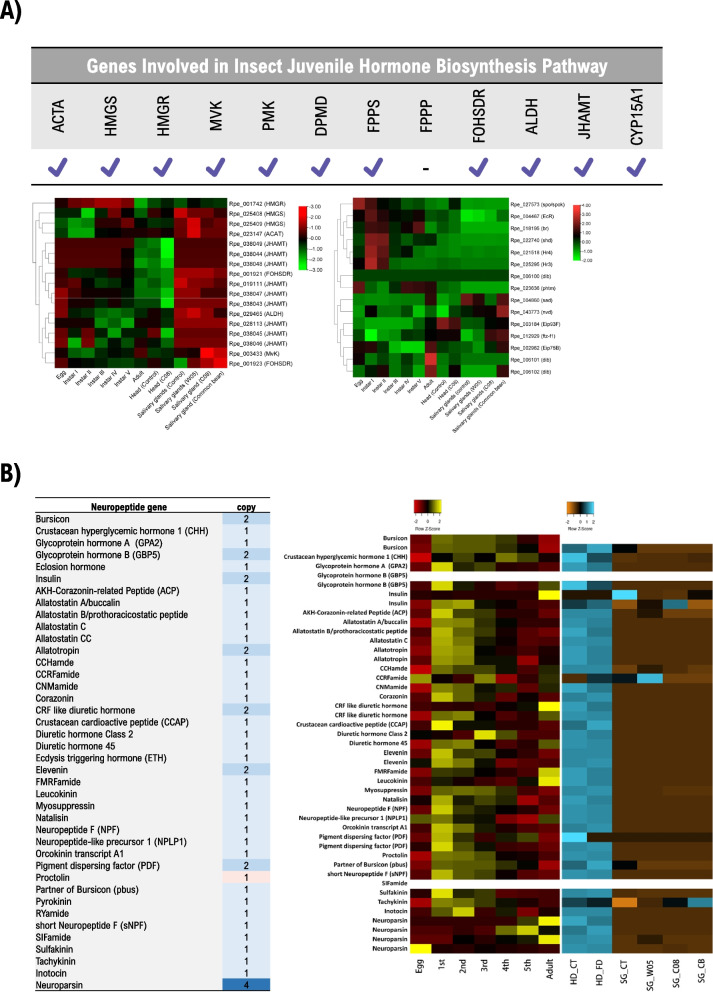
**A** Presence/absence of sesquiterpenoids pathway genes in *R. pedestris* (upper middle panel); gene expression (row Z-score) of sesquiterpenoid (lower left panel) and ecdysteroid (lower right panel) biosynthesis pathway genes across different developmental stages (egg/embryo, instars I to V and adult) and bean seed feeding (cultivated and wild soybeans C08, W05, respectively and common bean) comprised of organs head and salivary glands. **B** Annotation and copy number of neuropeptide genes in *R. pedestris* (left panel); gene expression (row Z-score) of neuropeptide genes across different developmental stages and upon bean seed feeding treatment (right panel)

Metamorphosis in insects is mainly regulated by two hormonal systems: (a) sesquiterpenoids – e.g., juvenile hormones (JHs) and (b) ecdysteroid hormones. The molecular diversity of relevant biosynthesis, receptor and response genes had important consequences that have led insects to adopt and adapt to a spectrum of temporal nuances in their life stages. The ability of ecdysteroid receptors to form homodimers and heterodimers, the presence/absence of duplication in JH receptor genes (*Met* and *gce*) and the chemical diversity of JH are among the notable differences between hemimetaboly and holometaboly. [77–82]. In this complex interplay of hormones, the anti-metamorphic actions of JH through the Met receptor activate *Kr-h1* which maintains the pre-metamorphic state. The precise periodicity of JH and ecdysteroid recapitulate an antagonistic interaction of their respective biosynthesis ensuring that a threshold size is reached before metamorphosis ensues [83]. In hemimetaboly, JH titer drops towards the end of the penultimate nymphal stage, while the same occurs for holometaboly but reappearing with Broad and Kr-h1 shortly before pupation. This is a process that ensures that development is modulated, that imaginal discs do not skip the pupal molt and that cell proliferation and patterning continues when 20E titer is high [84, 85]. Reminiscent to what has been described by [82], a status quo molt occurs in *Rhodnius prolixus* (Hemiptera) during the fourth-to-fifth instar intermolt where JH and 20E are co-occurring in contrast to what occurs during a progressive molt [86]. Concomitantly, the increase in 20E titer activates the Ashburner cascade (early, early/late and late genes) that are common to larval, pupal and adult molts [82]. Overall, the sesquiterpenoid hormones such as juvenile hormone is recognized as one of the most important regulators of development in insects [87], and identifying the members of the sesquiterpenoid gene family in *R. pedestris* is expected to serve as reference and resource to facilitate developmental and endocrinal studies. Besides the salivary glands, the brain’s corpus allatum (CA) is an important organ that responds to stress (e.g., starvation) via a mechanism of cross talk between insulin/IGF-1 and JH signaling [88]. Most of *R. pedestris*’ sesquiterpenoid biosynthesis pathway genes were identified except for *farnesyl diphosphate phosphatase* (*FPPase*) [89] (Fig. 2A, Supplementary Table 4). RNA-seq revealed distinct expression profiles for these genes across developmental stages (Fig. 2A, Supplementary Table 5). While multiple gene copies of *juvenile hormone acid methyltransferase* (*JHAMT*) were identified, Rpe_019111 exhibited relatively higher expression across development peaking at adult stage (Fig. 2A). In addition, expression levels of sesquiterpenoid pathway genes in the head and in the salivary glands based on different diets were analyzed (Fig. 2A, Supplementary Table 6) with *JHAMT* (Rpe_019111) in the head showing an apparent increased in gene expression relative to control. This expression was validated by RT-qPCR (Supplementary Fig. 26). A similar approach was performed for the ecdysteroid hormone biosynthesis genes where all genes (*neverland* – *nvd*, *spook/spookier* – *spo*/*spok, phantom* –* phtm, disembodied* – *dib*, *shadow* – *sad* and *shade* – *shd*) were identified in the genome (Fig. 2A). Notably, *dib* has three (3) copies in the genome with two (2) copies (Rpe_006101 and Rpe_006102) exhibiting expression across developmental stages. In addition, the *Ecdysone receptor* (*EcR*) together with early and late response genes *broad* (*br*), *Ecdysone-induced protein 75B* (*Eip75B*), *Hormone receptor 3* (*Hr3*), *Hormone receptor 4* (*Hr4*), *ftz transcription factor 1* (*ftz-f1*) and *Ecdysone-induced protein 93F* (*Eip93F*) orthologs were identified as single-copy genes in the genome (Fig. 2A). *Ecdysone-induced protein 74EF* (*Eip74EF*) was not identified in the genome.

### Neuropeptide hormones

Conserved arthropod neuropeptides were annotated from the *R. pedestris* genome, and their expression levels were examined across different development stages with RNA-seq data. There were in total 51 neuropeptide genes (1–2 copies each) annotated in *R. pedestris* (Fig. 2B; Supplementary Table 7). Trissin was not identified in the genome, and even with the presence of allatostatin C, allatostatin CC was not found in the vicinity and in other regions of the genome. Most of the annotated neuropeptide genes only have one (1) copy, while bursicon, glycoprotein hormone B (GBP5), insulin, allatotropin, CRF like diuretic hormone, elevenin and pigment dispersing factor (PDF) contained multiple copies. Except for insulin, duplicated copies (paralogs) of other neuropeptides are located next to each other, suggesting potential tandem duplication in generating these copies. A total of four tandem duplicated neuroparsin were also found in *R. pedestris* genome (Fig. 2B; Supplementary Table 7, Supplementary Fig. 2), which contrasts with most insects possessing only a single copy of neuroparsin gene where multiple transcripts are generated through alternative splicing to regulate sexual development and metamorphosis [90–93]. Among four (4) neuroparsin genes, one (1) copy had a higher expression level at the egg/embryo stage, while other three (3) copies showed highest expression levels at instar V and adult stages (Fig. 2B). Generally, neuropeptide genes were found to have higher expression levels at the onset of development (egg/embryo) gradually decreasing during development further suggesting crucial roles for these neuropeptides in early development (embryogenesis). Conversely, insulin, CRF like diuretic hormone, FMRFamide, leucokinin and neuroparsin had higher expression levels at the adult stage suggesting potential roles in adult physiology and reproduction (Fig. 2B,Supplementary Table 8). Among the annotated neuropeptides, glycoprotein hormone B (GBP5) and SIFamide were neither found to be expressed across different developmental stages nor in bean-feeding experiments. Meanwhile, insulin, CCRFamide, tachykinin and PDF were differentially expressed in the salivary gland suggesting potential roles in bean digestion and feeding physiology (Fig. 2B,Supplementary Table 9). Whether these neuropeptides could be further developed as targets for controlling the metabolism of *R. pedestris* requires further investigation.

### miRNAs

**Fig. 3:**
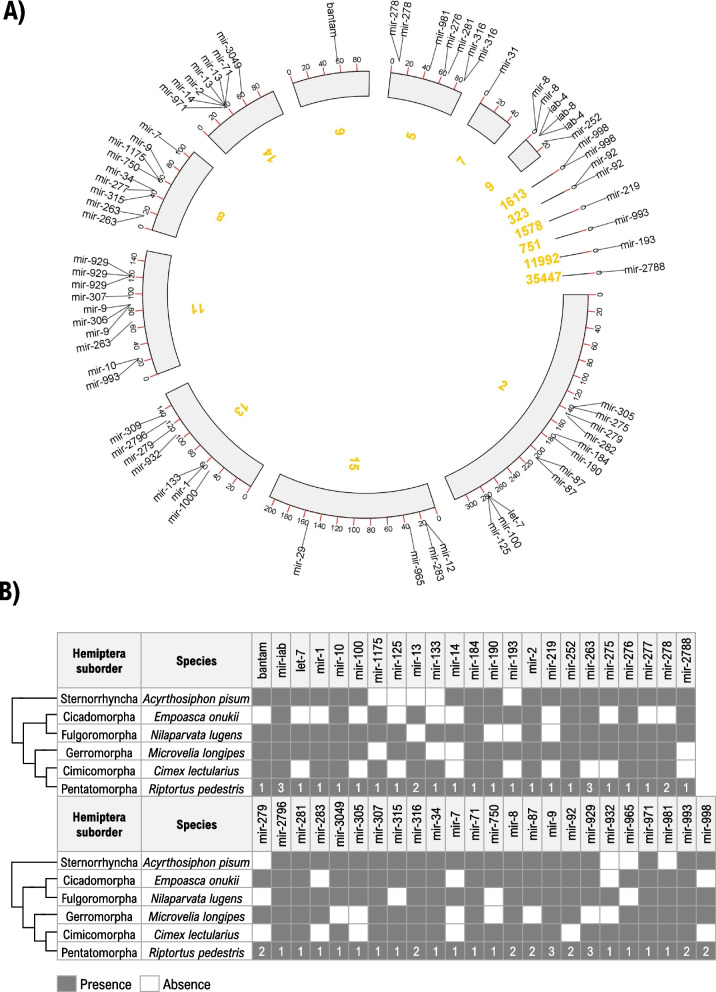
**A** miRNAs and their respective locations on the genome (scaffolds). **B** Presence/absence of predicted miRNAs on various hemipteran species

**Fig. 4:**
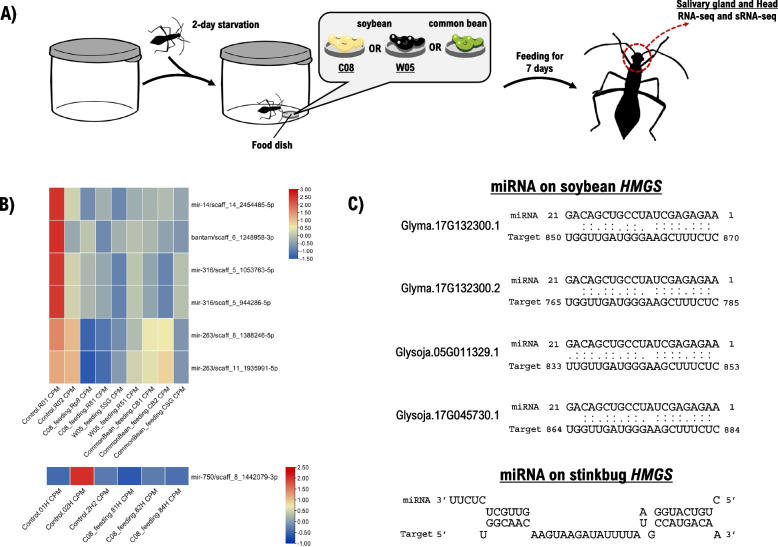
**A** Schematic diagram of seed feeding experimental setup. **B** Differentially expressed miRNAs in salivary gland (upper) and head (lower) upon feeding. **C** miRNA-281:*HMGS* binding structure. Binding structure of miRNA:soybean *HMGS* is retrieved from psRNATarget; binding structure of miRNA:stinkbug *HMGS* is retrieved from RNAhybrid

miRNAs are important posttranscriptional regulators of many biological processes in metazoans [94, 95]. Hence, they deserve an in-depth attention to understand the biology and ecology of insects. In *R. pedestris*, a total of 71 miRNAs were confidently annotated in sRNA-seq data which are also identified in other hemipteran genomes (Fig. 3, Supplementary Tables 10 and 11). Some of the miRNAs have multiple copies, including iab-4, iab-8, miR-13, miR-263, miR-278, miR-279, miR-316, miR-8, miR-87, miR-9, miR-92, miR-929, miR-993 and miR-998 (Supplementary Fig. 3). Other non-coding RNAs including but not limited to miRNA (i.e., rRNA, tRNA, snRNA, snoRNA and piRNA) were also predicted (Supplementary Figs. 4–23) [96, 97]. The gene expression of miRNAs as a function of diet was analyzed through the sRNA-seq data for both the salivary glands and the head (Fig. 4A, Supplementary Table 13). Differential expression analysis identified several microRNAs displaying differential expressions. These include bantam, miR-14, miR-316, miR-263 in the salivary glands (Fig. 4B upper) and miR-750 in the head (Fig. 4B, Supplementary Table 14). Expressed miRNAs in all (biological replicates) feeding groups were selected for gene target prediction analysis (Supplementary Table 11); and among these miRNAs, miR-281 was predicted to interact with *HMGS* in both stinkbug and soybean through its opposite arms designated as miR-281-5p and miR-281-3p (Fig. 4C).

## Discussion

Herbivores pose severe challenges on plants, and investigations on interaction between plants and herbivores (e.g., insects) will benefit the development of applications for pest management. The soybean pod-sucking stinkbug *R. pedestris* represents a major insect pest in Asia. In this study, we have provided not only its high-quality genome assembly but also its transcriptomes covering different developmental stages as resources. Furthermore, preliminary analysis of the relevant insect hormone biosynthesis pathways and neuropeptides serves as focal points that can further studies or investigations to address immediate issues on pest management. Thus, the resources that we present in this study are expected to not only provide knowledge on the biology and ecology or *R. pedestris* but also insights that can be used to develop species-specific pest management and mitigation strategies.

Sesquiterpenoid hormones such as juvenile hormones are important regulators of insect development and reproduction and have long been studied as a target for development of insecticides. In this study, we have not only analyzed the sesquiterpenoid pathway genes, but also found that the Jhamt enzyme, which is involved in the rate-determining step for the hormone synthesis, was differentially expressed in the salivary gland. This finding is congruent with previous studies on the crosstalk between insulin and juvenile hormone in beetles [98]; and in mosquitoes, [88] observed that starvation decreased JH synthesis via a decrease in insulin signaling in the CA while upregulating the expression of insulin receptor priming the gland to respond rapidly to increase in insulin levels. The dynamics on how *jhamt* and other sesquiterpenoid hormone pathway genes participate in insect physiology and soybean feeding warrants further investigation.

Neuropeptides are neurohormones and/or neuromodulators involved in the regulation of development, growth, reproduction, metabolism and behavior of insects. As such, important consideration is given to these peptides as potential targets for pest management [99, 100]. It is still unclear, however, what the actual number of functional neuropeptides there should be in each insect species. This is largely due to the varying number of reported neuropeptide genes across insect species, and the ability of one (1) preprohormone gene to generate functional neuropeptide paracopies further contributes to the neuropeptide diversity. For instance, 23 paracopies of functional peptides can be produced from a single FMRFamide gene in the cockroach *Periplaneta americana* [99, 101]. While transcriptomic and peptidomic approaches can analyze patterns and functions of the expressed neuropeptides, a high-quality genome offers the possibility of accounting the actual number of neuropeptide genes (including both putative and expressed) in the expression network. In holometabolans such as *Drosophila spp.*, *Anopheles gambiae*, *Aedes aegypti*, *Bombyx mori,* and *Apis mellifera*, an average of 40 neuropeptide genes could be identified in each species [102–106]. While the most complete annotated list of neuropeptides is mostly found in these holometabolan models than that of hemipterans, reported neuropeptides in insect pests are scarce. The annotation efforts on hemipteran neuropeptides have recently tackled this situation including species such as *Acyrthosiphon pisum*, *Rhodnius prolixus*, *Nilaparvata lugens*, *Diaphorina citri*, *Aphis craccivora* and *Daktulosphaira vitifoliae*, [107–112]. In this study, the genome has facilitated the identification of neuropeptides in *R. pedestris* which are found in holometabolans and in hemipterans such as *Acyrthosiphon pisum*, *Rhodnius prolixus*, *Nilaparvata lugens*, *Diaphorina citri*, *Aphis craccivora* and *Daktulosphaira vitifoliae*, [107–112]. Interestingly, Trissin and allatostatin CC were not found in the genome. Trissin was first discovered in the *Drosophila*’s brain and thoracicoabdominal ganglion and was later found in very small and restricted regions of the brain in *Bombyx mori* [113, 114]. There are reports showing the presence of trissin in crustaceans including crayfish and spider mites [115, 116]. While it is a conserved insect neuropeptide, trissin was also reported to be absent in hemipteran genomes [107], and this suggests a hemipteran-specific lineage loss of trissin. Finally, allatostatin CC is paralogous to allatostatin C (arthropod somatostatins) where they are hypothesized to have originated from tandem gene duplication [117]. Despite allatostatin C being successfully annotated in the *R. pedestris* genome, no allatostatin CC was found in the vicinity and in the other regions of the genome.

miRNAs have been considered as targets for controlling crop pests. For instance, rice expressing insect miRNAs resulted in developmental defects of the striped stem borer *C. suppressalis* [118, 119]. The miRNA repertoire of *R. pedestris* was first analyzed in this study, and several microRNAs were found to be differentially expressed in the salivary gland and in the head. For instance, miR-14 exhibited a decrease in gene expression in the salivary gland of the feeding group (Fig. 4B). This was an expected response to starvation. In *Drosophila*, miR-14 is involved in tissue-specific autophagy of salivary glands by downregulating *ip3k2* while increasing the production of IP3. IP3 together with calcium and calmodulin promote autophagy to maintain cellular homeostasis in response to stress such as starvation [120]. Furthermore, it was reported by [121] that miR-14 suppressed cell death in *Drosophila* induced by different stress stimuli. While miR-14 is dispensable, animals lacking it have reduced fitness (e.g., lifespan). This highlights the possibility of exploiting this physiological response to stress to reduce the fitness of *R. pedestris*. For example, there could be long non-coding RNAs (lncRNAs) acting as miRNA sponges that could reverse the physiological role of miR-14 in *R*. *pedestris*.

Cross-kingdom modulation of physiology by plant-derived and insect-derived microRNAs on plant–insect interactions were reported [122–126]. Here, we have shown that miR-281 could potentially target *HMGS*, a gene in mevalonate biosynthesis pathway, in both *R. pedestris* and soybean through opposite arms miR-281-5p and miR-281-3p, respectively. This is an intriguing observation that warrants subsequent validation. Gharehdaghi et al. [127] demonstrated that miRNAs from sunflower and sedr plants could be transmitted into the midgut of honeybee on feeding – suggesting essential roles of this phenomenon in mediating plant–insect interactions. Moreover, the target genes that the miRNAs are predicted to regulate are largely involved in development (e.g., hippo signaling, Wnt signaling and N-glycan biosynthesis pathways among others). Taking this further, [128] reported plant-derived miRNAs transmitted into the gut were detected in *Plutella*’s hemolymph suggesting a wider scale of possible physiological influence on the insect pest beyond its dietary response. Both these related studies have demonstrated the cross-kingdom regulation of physiological processes occur between plants and insects. Similarly, this study suggests the potential implication of the interaction between *Riptortus* and soybean through miR-281. Corollary to the related studies, the coexistence of this miRNA between the plant and insect provides evidence that transmission is plausible, while its role in developmental regulation is possibly acting through miRNA’s arm switching mechanism subject to experimental validation and confirmation. Interestingly, terpenoid biosynthesis in plants share the same upstream mevalonate biosynthesis pathway with sesquiterpenoid hormones biosynthesis in insects [129]. miR-281 is implicated in virus-host interaction, regulation of insect development and insecticide resistance. miR-281 can be found in several mosquito species such as *An. gambiae*, *Ae. aegypti*, *Cu. quinquefasciatus*, *An. stephensi*, and *Ae. albopictus* [130]. Specifically, in *Ae. albopictus*, miR-281 was demonstrated to be specifically abundant in the midgut facilitating the replication of dengue virus [131]. Furthermore, endogenous or exogenous miR-281 was demonstrated to regulate insect development as in the case of *B. mori* where miR-281 targets specific isoform of *EcR-B* to regulate development, and its expression is only suppressed by 20E but not JH [132]. In *Ae. aegypti*, miR-281 is the most abundant in the gut, and it is antagonistically regulated by Met (induction) and EcR (repression)-mediated signaling pathway [133]. Moreover, studies in different insects indicate its expression dynamically change across development. In *Thrips tabaci* Lindeman, compared to larval stages, it shows higher expression in pupal and adult stages [134]. Furthermore, in *Plutella xylostella*, its *EcR* expression can be reduced by Cve-miR-281-3p, resulting in the delay of the rise of *EcR*, growth and pupation during miRNA agomir treatment [112]. This interaction between miR-281 and *EcR* is also shown in *B. mori*, which implies that the interaction between miR-281 and *EcR* may be conserved in Lepidoptera insects. In addition, previous study reported miR-281–1-5p conferred the house fly, *Musca domestica* propoxur resistance by targeting CYP6G4 [135].

## Conclusion

With the data resource generated in this study, we have established *R. pedestris*, an emerging insect pest, as a laboratory model for studying plant–insect interactions. We have also analyzed key regulators of their development, physiology, and reproduction and provided key insights on how they could be of potential use as targets of pest control agents.

### Supplementary Information

**Supplementary Material 1. **

**Supplementary Material 2.**

## Data Availability

The genome assembly is deposited in NCBI with accession JACJUX000000000, and the raw reads generated in this study are deposited in NCBI database under BioProject accession no. PRJNA576936. The genome annotation files are deposited in the Figshare (https://doi.org/10.6084/m9.figshare.22338193). All other data will be provided upon reasonable request.

## References

[1] Do Bae S, Kim HJ, Mainali BP (2014). Infestation of Riptortus pedestris (Fabricius) decreases the nutritional quality and germination potential of soybean seeds. Journal of Asia-Pacific Entomology.

[2] Kobayashi T (1972). Biology of insect pests of soybean and their control. Japan Agricultural Research Quarterly.

[3] Painter RH (1951). Insect resistance in crop plants. LWW.

[4] Stout MJ. Chapter 1 - Host-Plant Resistance in Pest Management. In: Abrol DP, editor. Integrated Pest Management. San Diego: Academic Press; 2014. p. 1–21. 10.1016/B978-0-12-398529-3.00002-6.

[5] Van Emden HF, Harrington R, editors. Aphids as crop pests. Cabi; 2017. 10.1079/9781780647098.000017.

[6] Smith CM, Chuang WP (2014). Plant resistance to aphid feeding: behavioral, physiological, genetic and molecular cues regulate aphid host selection and feeding. Pest Manag Sci.

[7] Dogimont C, Bendahmane A, Chovelon V, Boissot N (2010). Host plant resistance to aphids in cultivated crops: genetic and molecular bases, and interactions with aphid populations. CR Biol.

[8] Smith CM (2005). Plant resistance to arthropods: molecular and conventional approaches.

[9] Hesler LS, Tilmon KJ, Varenhorst AJ, Conzemius SR, Taliercio E, Beckendorf EA (2022). Challenges and Prospects of Wild Soybean as a Resistance Source Against Soybean Aphid (Hemiptera: Aphididae). Ann Entomol Soc Am.

[10] Bansal R, Michel A (2015). Molecular adaptations of aphid biotypes in overcoming host-plant resistance. Short Views on Insect Genomics and Proteomics: Insect Genomics.

[11] Wenger JA, Cassone BJ, Legeai F, Johnston JS, Bansal R, Yates AD, Michel A (2020). Whole genome sequence of the soybean aphid, Aphis glycines. Insect Biochem Mol Biol.

[12] Yates-Stewart AD, Daron J, Wijeratne S, Shahid S, Edgington HA, Slotkin RK, Michel A (2020). Soybean aphids adapted to host-plant resistance by down regulating putative effectors and up regulating transposable elements. Insect Biochem Mol Biol.

[13] Rahman MM, Lim UT (2017). Evaluation of mature soybean pods as a food source for two pod-sucking bugs, Riptortus pedestris (Hemiptera: Alydidae) and Halyomorpha halys (Hemiptera: Pentatomidae). PLoS ONE.

[14] Li K, Zhang X, Guo J, Penn H, Wu T, Li L, Han T (2019). Feeding of Riptortus pedestris on soybean plants, the primary cause of soybean staygreen syndrome in the Huang-Huai-Hai river basin. The Crop Journal..

[15] Li W, Gao Y, Hu Y, Chen J, Zhang J, Shi S (2021). Field cage assessment of feeding damage by Riptortus pedestris on soybeans in China. Insects.

[16] Yan X, An J, Gao X (2021). Relationship between population density of Riptortus pedestris and spring sowing soybean yiled using cumulative insect-days method. Plant Prot.

[17] Zhang H, Wang Y, Wang Z, Ding W, Xu K, Li L, Huang X (2022). Modelling the current and future potential distribution of the bean bug Riptortus pedestris with increasingly serious damage to soybean. Pest Manag Sci.

[18] Mainali BP, Kim HJ, Yoon YN, Oh IS, Do Bae S (2014). Evaluation of different leguminous seeds as food sources for the bean bug Riptortus pedestris. J Asia Pac Entomol.

[19] McPherson RM, Newsom LD, Farthing BF (1979). Evaluation of four stink bug species from three genera affecting soybean yield and quality in Louisiana. Journal of economic entomology.

[20] Hobbs HA, Hill CB, Grau CR, Koval NC, Wang Y, Pedersen WL, Hartman GL (2006). Green stem disorder of soybean. Plant dis.

[21] Walling LL (2008). Avoiding effective defenses: strategies employed by phloem-feeding insects. Plant Physiol.

[22] Kikuchi Y, Hosokawa T, Fukatsu T (2011). Specific developmental window for establishment of an insect-microbe gut symbiosis. Appl Environ Microbiol.

[23] Lee SJ, Yang YT, Kim S, Lee MR, Kim JC, Park SE, Kim JS (2019). Transcriptional response of bean bug (Riptortus pedestris) upon infection with entomopathogenic fungus, Beauveria bassiana JEF-007. Pest management science..

[24] Chaudhary R, Atamian HS, Shen Z, Briggs SP, Kaloshian I (2014). GroEL from the endosymbiont Buchnera aphidicola betrays the aphid by triggering plant defense. Proc Natl Acad Sci.

[25] Elzinga DA, De Vos M, Jander G (2014). Suppression of plant defenses by a Myzus persicae (green peach aphid) salivary effector protein. Mol Plant Microbe Interact.

[26] Kettles GJ, Kaloshian I (2016). The potato aphid salivary effector Me47 is a glutathione-S-transferase involved in modifying plant responses to aphid infestation. Front Plant Sci.

[27] Chen C, Chen H, Zhang Y, Thomas HR, Frank MH, He Y, Xia R (2020). TBtools: An Integrative Toolkit Developed for Interactive Analyses of Big Biological Data. Mol Plant.

[28] Du M, Wang Y, Chen C, Li X, Feng R, Zhou X, Yang X (2022). Molecular characterization and pathogenicity of a novel soybean-infecting monopartite geminivirus in China. Viruses.

[29] Gong C, Liu Y, Ma Y, Zhan X, Zhou Z, Zhu X, et al. Influence of electrostatic spraying on drift and deposition distribution. J. Sichuan Agric. Univ. 2022;40(02):220–226 + 242. 10.16036/j.issn.1000-2650202109029.

[30] Prajapati A, Singh RP, Kumar B, Kewat RN (2020). Physical and biochemical studies of lentil (Lens culinaris Medik.) varieties. Int J Curr Microbiol Appl Sci.

[31] Xu M, Guo L, Gu S, Wang O, Zhang R, Fan G, Liu X. TGS-GapCloser: fast and accurately passing through the Bermuda in large genome using error-prone third-generation long reads. BioRxiv. 2019;831248. 10.1101/831248

[32] Dong Y, Shen D, Dou D, Xia A. Characterization of salivary secreted proteins that induce cell deathfrom Riptortus pedestris (Fabricius) and their roles in insect-plant interactions. Front Plant Sci. 2022;13:912603. 10.3389/fpls.2022.912603. 10.3389/fpls.2022.912603 PMC9289560 35860545

[33] Fu S, Duan Y, Wang S, Ren Y, Bu W (2021). Comparative transcriptomic analysis of Riptortus pedestris (Hemiptera: Alydidae) to characterize wing formation across all developmental stages. Insects.

[34] Li C, Wong AY, Wang S, Jia Q, Chuang WP, Bendena WG, Hui JH (2018). miRNA-mediated interactions in and between plants and insects. International Journal of Molecular Sciences..

[35] Huang HJ, Ye YX, Ye ZX, Yan XT, Wang X, Wei ZY, Chen JP, Li JM, Sun ZT, Zhang CX (2021). Chromosome-level genome assembly of the bean bug Riptortus pedestris. Mol Ecol Resour.

[36] Weisenfeld NI, Kumar V, Shah P, Church DM, Jaffe DB (2017). Direct determination of diploid genome sequences. Genome Res.

[37] Putnam NH, O'Connell BL, Stites JC, Rice BJ, Blanchette M, Calef R, Green RE (2016). Chromosome-scale shotgun assembly using an in vitro method for long-range linkage. Genome research..

[38] Dudchenko O, Batra S. S, Omer A. D, Nyquist S. K, Hoeger M, Durand N. C, De Aiden E. L (2017). novo assembly of the Aedes aegypti genome using Hi-C yields chromosome-length scaffolds. Science..

[39] Durand NC, Shamim MS, Machol I, Rao SSP, Huntley MH, Lander ES, Aiden EL (2016). Juicer Provides a One-Click System for Analyzing Loop-Resolution Hi-C Experiments. Cell Syst.

[40] Salmela L, Rivals E (2014). Sequence analysis LoRDEC: accurate and efficient long read error correction.

[41] Du H, Yu Y, Ma Y, Gao Q, Cao Y, Chen Z, Liang C (2017). Sequencing and de novo assembly of a near complete indica rice genome. Nature Communications..

[42] Simão FA, Waterhouse RM, Ioannidis P, Kriventseva EV, Zdobnov EM (2015). BUSCO: assessing genome assembly and annotation completeness with single-copy orthologs. Bioinformatics.

[43] Soderlund C, Bomhoff M, Nelson WM (2011). SyMAP v3 4: a turnkey synteny system with application to plant genomes. Nucleic acids research..

[44] Soderlund C, Nelson W, Shoemaker A, Paterson A (2006). SyMAP: A system for discovering and viewing syntenic regions of FPC maps. Genome Res.

[45] Baril T, Galbraith JG, Hayward A (2023). Earl Grey: a fully automated user-friendly transposable element annotation and analysis pipeline.

[46] Jonathan M. Palmer, & Jason Stajich. Funannotate v1.8.1: Eukaryotic genome annotation (v1.8.1). Zenodo. 2020. 10.5281/zenodo.4054262.

[47] Lomsadze A, Ter-Hovhannisyan V, Chernoff YO, Borodovsky M (2005). Gene identification in novel eukaryotic genomes by self-training algorithm. Nucleic Acids Res.

[48] Haas BJ, Salzberg SL, Zhu W, Pertea M, Allen JE, Orvis J, Wortman JR (2008). Automated eukaryotic gene structure annotation using EVidenceModeler and the Program to Assemble Spliced Alignments. Genome biology..

[49] Stanke M, Keller O, Gunduz I, Hayes A, Waack S, Morgenstern B (2006). AUGUSTUS: ab initio prediction of alternative transcripts. Nucleic acids research..

[50] Majoros WH, Pertea M, Antonescu C, Salzberg SL (2003). GlimmerM, Exonomy and Unveil: three ab initio eukaryotic genefinders. Nucleic Acids Res.

[51] Korf I (2004). Gene finding in novel genomes. BMC Bioinformatics.

[52] Buchfink B, Reuter K, Drost HG (2021). Sensitive protein alignments at tree-of-life scale using DIAMOND. Nat Methods.

[53] Shumate A, Wong B, Pertea G, Pertea M (2022). Improved transcriptome assembly using a hybrid of long and short reads with StringTie. PLoS Comput Biol.

[54] Law ST, Nong W, So WL, Baril T, Swale T, Chan CB, Hui JH (2022). Chromosomal-level reference genome of the moth Heortia vitessoides (Lepidoptera: Crambidae), a major pest of agarwood-producing trees. Genomics..

[55] Emms DM, Kelly S (2019). OrthoFinder: phylogenetic orthology inference for comparative genomics. Genome Biol.

[56] Katoh K, Standley DM (2013). MAFFT multiple sequence alignment software version 7: improvements in performance and usability. Mol Biol Evol.

[57] Minh BQ, Schmidt HA, Chernomor O, Schrempf D, Woodhams MD, Von Haeseler A, Lanfear R (2020). IQ-TREE 2: new models and efficient methods for phylogenetic inference in the genomic era. Mol Biol Evol.

[58] Kanehisa M (2019). Toward understanding the origin and evolution of cellular organisms. Protein Sci.

[59] Kanehisa M, Goto S. KEGG: kyoto encyclopedia of genes and genomes. Nucleic Acids Res. 2000;28(1):27–30. 10.1093/nar/28.1.27 PMC102409 10592173

[60] Kanehisa M, Furumichi M, Sato Y, Kawashima M, Ishiguro-Watanabe M (2023). KEGG for taxonomy-based analysis of pathways and genomes. Nucleic Acids Res.

[61] Gramates LS, Agapite J, Attrill H, Calvi BR, Crosby MA, Dos Santos G, Strelets VB (2022). FlyBase: a guided tour of highlighted features. Genetics..

[62] Chen Y, Singh A, Kaithakottil G. G, Mathers T. C, Gravino M, Mugford S. T, Hogenhout S. A (2020). An aphid RNA transcript migrates systemically within plants and is a virulence factor. Proceedings of the National Academy of Sciences..

[63] Kumar S, Stecher G, Tamura K (2016). MEGA7: Molecular evolutionary genetics analysis version 7.0 for bigger datasets. Mol Biol Evol.

[64] Dyrløv BJ, Nielsen H, von Heijne G, Brunak S (2004). Improved prediction of signal peptides: SignalP 3.0. J Mol Biol.

[65] Veenstra JA (2000). Mono-and dibasic proteolytic cleavage sites in insect neuroendocrine peptide precursors. Archives of Insect Biochemistry and Physiology: Published in Collaboration with the Entomological Society of America.

[66] Chen B, Teh BS, Sun C, Hu S, Lu X, Boland W, Shao Y (2016). Biodiversity and activity of the gut microbiota across the life history of the insect herbivore Spodoptera littoralis. Sci Rep.

[67] Friedländer MR, Mackowiak SD, Li N, Chen W, Rajewsky N (2012). miRDeep2 accurately identifies known and hundreds of novel microRNA genes in seven animal clades. Nucleic Acids Res.

[68] Kozomara A, Birgaoanu M, Griffiths-Jones S (2019). miRBase: from microRNA sequences to function. Nucleic Acids Res.

[69] Fromm B, Høye E, Domanska D, Zhong X, Aparicio-Puerta E, Ovchinnikov V, Peterson K. J (2022). MirGeneDB 2. 1: toward a complete sampling of all major animal phyla. Nucleic acids res.

[70] Powell D (2015). Degust: visualize, explore and appreciate RNA-seq differential gene-expression data. COMBINE RNA-seq workshop.

[71] Joshi T, Wang J, Zhang H, Chen S, Zeng S, Xu B, Xu D (2017). The evolution of soybean knowledge base (SoyKB). Plant Genomics Databases.

[72] Dai X, Zhuang Z, Zhao PX (2018). psRNATarget: a plant small RNA target analysis server (2017 release). Nucleic Acids Res.

[73] Enright A, John B, Gaul U, Tuschl T, Sander C, Marks D (2003). MicroRNA targets in Drosophila. Genome Biol.

[74] Krüger J, Rehmsmeier M (2006). RNAhybrid: microRNA target prediction easy, fast and flexible. Nucleic acids research..

[75] Mei Y, Jing D, Tang S, Chen X, Chen H, Duanmu H (2022). InsectBase 2.0: a comprehensive gene resource for insects. Nucleic acids res.

[76] Hasebe M, Shiga S (2022). Clock gene-dependent glutamate dynamics in the bean bug brain regulate photoperiodic reproduction. PLoS Biol.

[77] Baumann A, Fujiwara Y, Wilson TG (2010). Evolutionary divergence of the paralogs Methoprene tolerant (Met) and germ cell expressed (gce) within the genus Drosophila. J Insect Physiol.

[78] Hill RJ, Billas IM, Bonneton F, Graham LD, Lawrence MC (2013). Ecdysone receptors: from the Ashburner model to structural biology. Annu Rev Entomol.

[79] Jindra M, Palli SR, Riddiford LM (2013). The juvenile hormone signaling pathway in insect development. Annu Rev Entomol.

[80] Konopova B, Jindra M (2007). Juvenile hormone resistance gene Methoprene-tolerant controls entry into metamorphosis in the beetle Tribolium castaneum. Proc Natl Acad Sci.

[81] Konopova B, Smykal V, Jindra M (2011). Common and distinct roles of juvenile hormone signaling genes in metamorphosis of holometabolous and hemimetabolous insects. PLoS ONE.

[82] Truman JW (2019). The Evolution of Insect Metamorphosis. Curr Biol.

[83] Liu S, Li K, Gao Y, Liu X, Chen W, Ge W, Li S (2018). Antagonistic actions of juvenile hormone and 20-hydroxyecdysone within the ring gland determine developmental transitions in Drosophila. PNAS.

[84] Champlin DT, Reiss SE, Truman JW (1999). Hormonal control of ventral diaphragm myogenesis during metamorphosis of the moth, Manduca sexta. Dev Genes Evol.

[85] Truman JW, Hiruma K, Allee JP, MacWhinnie SGB, Champlin DT, Riddiford L (2006). Juvenile hormone is required to couple imaginal disc formation with nutrition in insects. Science.

[86] Villalobos-Sambucaro MJ, Nouzova M, Ramirez CE, Eugenia Alzugaray M, Fernandez-Lima F, Ronderos JR, Noriega FG (2020). The juvenile hormone described in Rhodnius prolixus by Wigglesworth is juvenile hormone III skipped bisepoxide. Sci Rep.

[87] Tsang SS, Law ST, Li C, Qu Z, Bendena WG, Tobe SS, Hui JH (2020). Diversity of insect sesquiterpenoid regulation. Front Genet.

[88] Perez-Hedo M, Rivera-Perez C, Noriega FG (2014). Starvation increases insulin sensitivity and reduces juvenile hormone synthesis in mosquitoes. PLoS ONE.

[89] Cheng D, Meng M, Peng J, Qian W, Kang L, Xia Q (2014). Genome-wide comparison of genes involved in the biosynthesis, metabolism, and signaling of juvenile hormone between silkworm and other insects. Genet Mol Biol.

[90] Badisco L, Claeys I, Van Loy T, Van Hiel M, Franssens V, Simonet G, Broeck JV (2007). Neuroparsins, a family of conserved arthropod neuropeptides. Gen Comp Endocrinol.

[91] Lenaerts C, Monjon E, Van Lommel J, Verbakel L, Broeck JV (2019). Peptides in insect oogenesis. Current opinion in insect science.

[92] Tanaka Y. Chapter 84 - Neuroparsin. In: Ando H, Ukena K, Nagata S, editors. Handbook of Hormones (Second Edition). 2nd Ed. San Diego: Academic Press; 2021. p. 761–3. 10.1016/B978-0-12-820649-2.00204-7.

[93] Veenstra JA (2010). What the loss of the hormone neuroparsin in the melanogaster subgroup of Drosophila can tell us about its function. Insect Biochem Mol Biol.

[94] Qu Z, Bendena WG, Tobe SS, Hui JH (2018). Juvenile hormone and sesquiterpenoids in arthropods: biosynthesis, signaling, and role of MicroRNA. J Steroid Biochem Mol Biol.

[95] Qu Z, Nong W, So WL, Barton-Owen T, Li Y, Leung TCN, Li C, Baril T, Wong AYP, Swale T, Chan TF, Hayward A, Ngai SM, Hui JHL (2020). Millipede genomes reveal unique adaptations during myriapod evolution. PLoS Biol.

[96] Gebert D, Hewel C, Rosenkranz D (2017). unitas: the universal tool for annotation of small RNAs. BMC Genomics.

[97] Kopylova E, Noé L, Touzet H (2012). SortMeRNA: fast and accurate filtering of ribosomal RNAs in metatranscriptomic data. Bioinformatics (Oxford, England).

[98] Xu J, Sheng Z, Palli SR (2013). Juvenile hormone and insulin regulate trehalose homeostasis in the red flour beetle, Tribolium castaneum. PLoS Genet.

[99] Altstein M, Nassel DR (2010). Neuropeptide signaling in insects. Neuropeptide Syst Targets Parasite Pest Control.

[100] Bendena WG (2010). Neuropeptide physiology in insects. Adv Exp Med Biol.

[101] Predel R, Neupert S, Wicher D, Gundel M, Roth S, Derst C (2004). Unique accumulation of neuropeptides in an insect: FMRFamide-related peptides in the cockroach. Periplaneta americana European Journal of Neuroscience.

[102] Boerjan B, Cardoen D, Bogaerts A, Landuyt B, Schoofs L, Verleyen P (2010). Mass spectrometric profiling of (neuro)-peptides in the worker honeybee. Apis mellifera Neuropharmacology.

[103] Hewes RS, Taghert PH (2001). Neuropeptides and neuropeptide receptors in the Drosophila melanogaster genome. Genome Res.

[104] Matthews BJ, McBride CS, DeGennaro M, Despo O, Vosshall LB (2016). The neurotranscriptome of the Aedes aegypti mosquito. BMC Genomics.

[105] Riehle MA, Garczynski SF, Crim JW, Hill CA, Brown MR (2002). Neuropeptides and peptide hormones in Anopheles gambiae. Science.

[106] Roller L, Yamanaka N, Watanabe K, Daubnerová I, Žitňan D, Kataoka H, Tanaka Y (2008). The unique evolution of neuropeptide genes in the silkworm Bombyx mori. Insect Biochem Mol Biol.

[107] Gao H, Li Y, Zhang H, Wang S, Feng F, Tang J, Li B (2023). Comparative study of neuropeptide signaling systems in Hemiptera. Insect Sci.

[108] Huybrechts J, Bonhomme J, Minoli S, Prunier-Leterme N, Dombrovsky A, Abdel-Latief M, Tagu D (2010). Neuropeptide and neurohormone precursors in the pea aphid. Acyrthosiphon pisum. Insect molecular biology..

[109] Li X, Du L, Jiang XJ, Ju Q, Qu CJ, Qu MJ, Liu TX (2020). Identification and characterization of neuropeptides and their G protein-coupled receptors (GPCRs) in the cowpea aphid Aphis craccivora. Front Endocrinol.

[110] Ons S, Sterkel M, Diambra L, Urlaub H, Rivera-Pomar R (2011). Neuropeptide precursor gene discovery in the Chagas disease vector Rhodnius prolixus. Insect Mol Biol.

[111] Tanaka Y, Suetsugu Y, Yamamoto K, Noda H, Shinoda T (2014). Transcriptome analysis of neuropeptides and G-protein coupled receptors (GPCRs) for neuropeptides in the brown planthopper Nilaparvata lugens. Peptides.

[112] Wang Z, Zhou W, Hameed MS, Liu J, Zeng X (2018). Characterization and expression profiling of neuropeptides and G-protein-coupled receptors (GPCRs) for neuropeptides in the Asian citrus psyllid, Diaphorina citri (Hemiptera: Psyllidae). Int J Mol Sci.

[113] Ida T, Takahashi T, Tominaga H, Sato T, Kume K, Yoshizawa-Kumagaye K, Kojima M (2011). Identification of the endogenous cysteine-rich peptide trissin, a ligand for an orphan G protein-coupled receptor in Drosophila. BBRC.

[114] Roller L, Čižmár D, Gáliková Z, Bednár B, Daubnerová I, Žitňan D (2016). Molecular cloning, expression and identification of the promoter regulatory region for the neuropeptide trissin in the nervous system of the silkmoth Bombyx mori. Cell Tissue Res.

[115] Veenstra JA (2015). The power of next-generation sequencing as illustrated by the neuropeptidome of the crayfish Procambarus clarkii. Gen Comp Endocrinol.

[116] Veenstra JA, Rombauts S, Grbić M (2012). In silico cloning of genes encoding neuropeptides, neurohormones and their putative G-protein coupled receptors in a spider mite. Insect Biochem Mol Biol.

[117] Veenstra JA (2009). Allatostatin C and its paralog allatostatin double C: the arthropod somatostatins. Insect Biochem Mol Biol.

[118] Jiang S, Wu H, Liu H, Zheng J, Lin Y, Chen H (2017). The overexpression of insect endogenous small RNAs in transgenic rice inhibits growth and delays pupation of striped stem borer (Chilo suppressalis). Pest Manag Sci.

[119] Liu H, Shen E, Wu H, Ma W, Chen H, Lin Y (2022). Trans-kingdom expression of an insect endogenous microRNA in rice enhances resistance to striped stem borer Chilo suppressalis. Pest Manag Sci.

[120] Nelson C, Ambros V, Baehrecke EH (2014). miR-14 regulates autophagy during developmental cell death by targeting ip3-kinase 2. Mol Cell.

[121] Xu P, Vernooy SY, Guo M, Hay BA (2003). The Drosophila microRNA Mir-14 suppresses cell death and is required for normal fat metabolism. Curr Biol.

[122] Bordoloi KS, Agarwala N (2021). MicroRNAs in plant insect interaction and insect pest control. Plant Gene.

[123] Dai Z, Tan J, Zhou C, Yang X, Yang F, Zhang S, Shi Z (2019). The OsmiR396–Os GRF 8–OsF3H-flavonoid pathway mediates resistance to the brown planthopper in rice (Oryza sativa). Plant Biotechnol J.

[124] Tan J, Wu Y, Guo J, Li H, Zhu L, Chen R, Du B (2020). A combined microRNA and transcriptome analyses illuminates the resistance response of rice against brown planthopper. BMC genomics..

[125] Wang ZZ, Ye XQ, Shi M, Li F, Wang ZH, Zhou YN, Gu QJ, Wu XT, Yin CL, Guo DH, Hu RM, Hu NN, Chen T, Zheng BY, Zou JN, Zhan LQ, Wei SJ, Wang YP, Huang JH, Fang XD, Chen XX (2018). Parasitic insect-derived miRNAs modulate host development. Nat Commun.

[126] Zhu K, Liu M, Fu Z, Zhou Z, Kong Y, Liang H, Chen X (2017). Plant microRNAs in larval food regulate honeybee caste development. PLoS Genet.

[127] Gharehdaghi L, Bakhtiarizadeh MR, He K, Harkinezhad T, Tahmasbi G, Li F (2021). Diet-derived transmission of MicroRNAs from host plant into honey bee Midgut. BMC Genomics.

[128] Zhang LL, Jing XD, Chen W, Wang Y, Lin JH, Zheng L, You MS (2019). Host plant-derived miRNAs potentially modulate the development of a cosmopolitan insect pest, Plutella xylostella. Biomolecules.

[129] Boncan DAT, Tsang SS, Li C, Lee IH, Lam HM, Chan TF, Hui JH (2020). Terpenes and terpenoids in plants: Interactions with environment and insects. Int J Mol Sci.

[130] Feng X, Zhou S, Wang J, Hu W (2018). microRNA profiles and functions in mosquitoes. PLoS Negl Trop Dis.

[131] Zhou Y, Liu Y, Yan H, Li Y, Zhang H, Xu J, Puthiyakunnon S, Chen X (2014). miR-281, an abundant midgut-specific miRNA of the vector mosquito Aedes albopictus enhances dengue virus replication. Parasit Vectors.

[132] Jiang J, Ge X, Li Z, Wang Y, Song Q, Stanley DW, Tan A, Huang Y (2013). MicroRNA-281 regulates the expression of ecdysone receptor (EcR) isoform B in the silkworm, Bombyx mori. Insect Biochem Mol Biol.

[133] Zhang X, Raikhel AS (2021). Hormonal regulation of microRNA expression dynamics in the gut of the yellow fever mosquito Aedes aegypti. RNA Biol.

[134] Balan RK, Ramasamy A, Hande RH, Gawande SJ, Krishna Kumar NK (2018). Genome-wide identification, expression profiling, and target gene analysis of microRNAs in the Onion thrips, Thrips tabaci Lindeman (Thysanoptera: Thripidae), vectors of tospoviruses (Bunyaviridae). Ecol Evol.

[135] You C, Zhang L, Song J, Zhang L, Zhen C, Gao X (2023). The variation of a cytochrome P450 gene, CYP6G4, drives the evolution of Musca domestica L (Diptera: Muscidae) resistance to insecticides in China. Int J Bio Macromol.

