# Supplementary material for: Elucidating the ecophysiology of soybean pod-sucking stinkbug Riptortus pedestris (Hemiptera: Alydidae) based on de novo genome assembly and transcriptome analysis
Source: BMC Genomics. 2024 Apr 2;25:327. doi: 10.1186/s12864-024-10232-2 (PMC10985886; doi:10.1186/s12864-024-10232-2)
Supplement: Supplementary file 1 — Supplementary Material 1. [file 12864_2024_10232_MOESM1_ESM.pptx]

## Slide 1
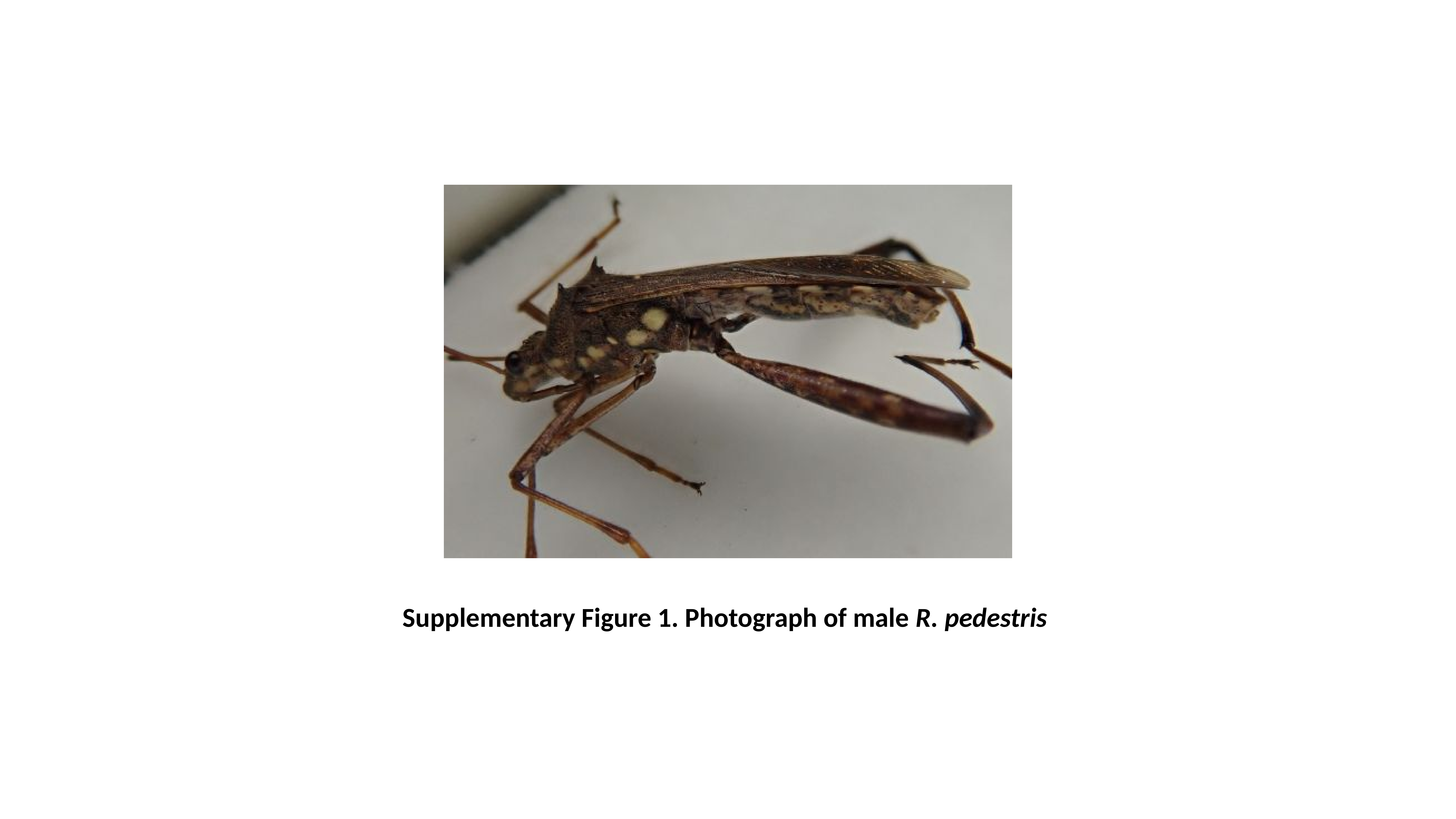

Supplementary Figure 1. Photograph of male R. pedestris

## Slide 2
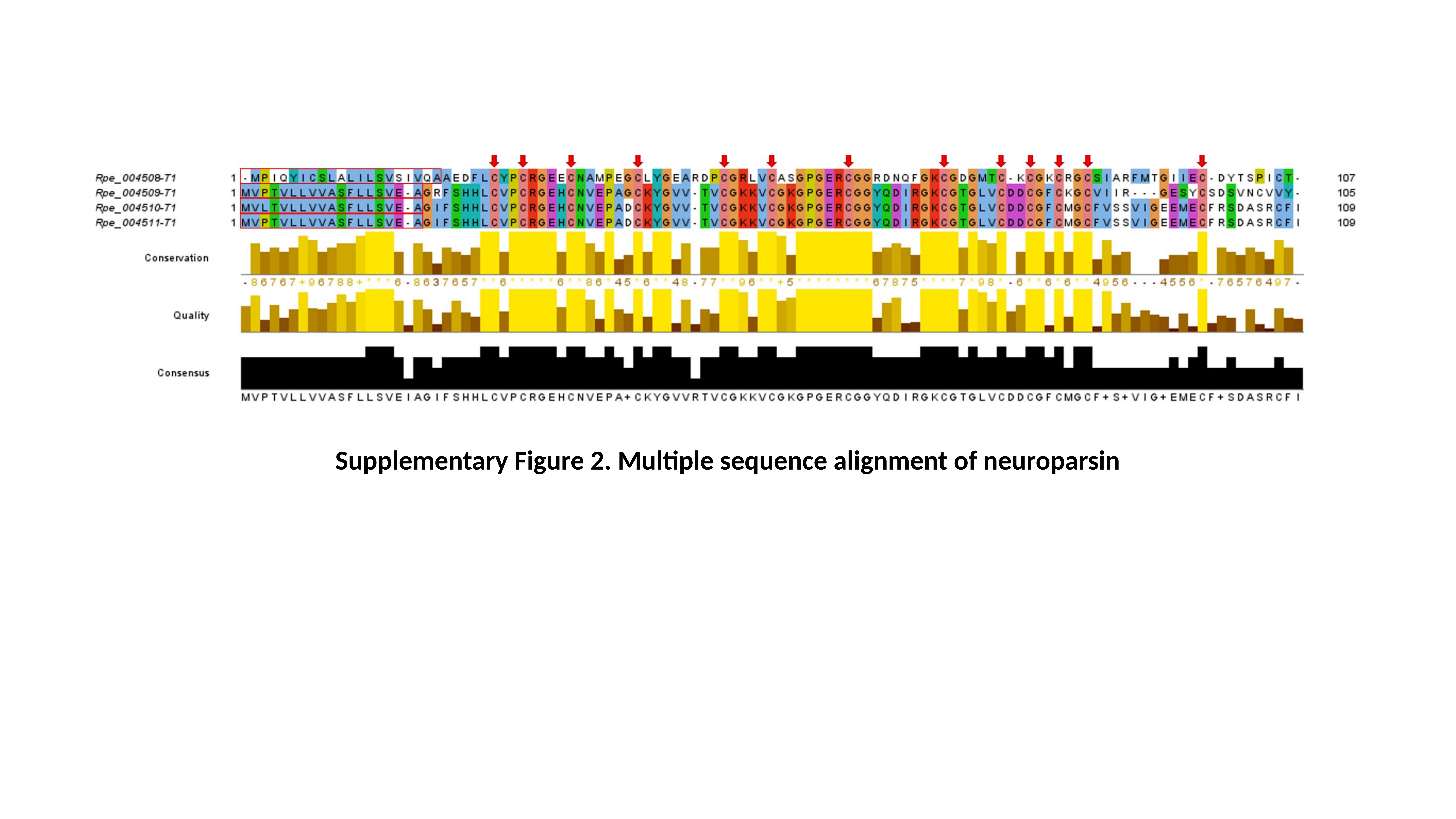

Supplementary Figure 2. Multiple sequence alignment of neuroparsin

## Slide 3
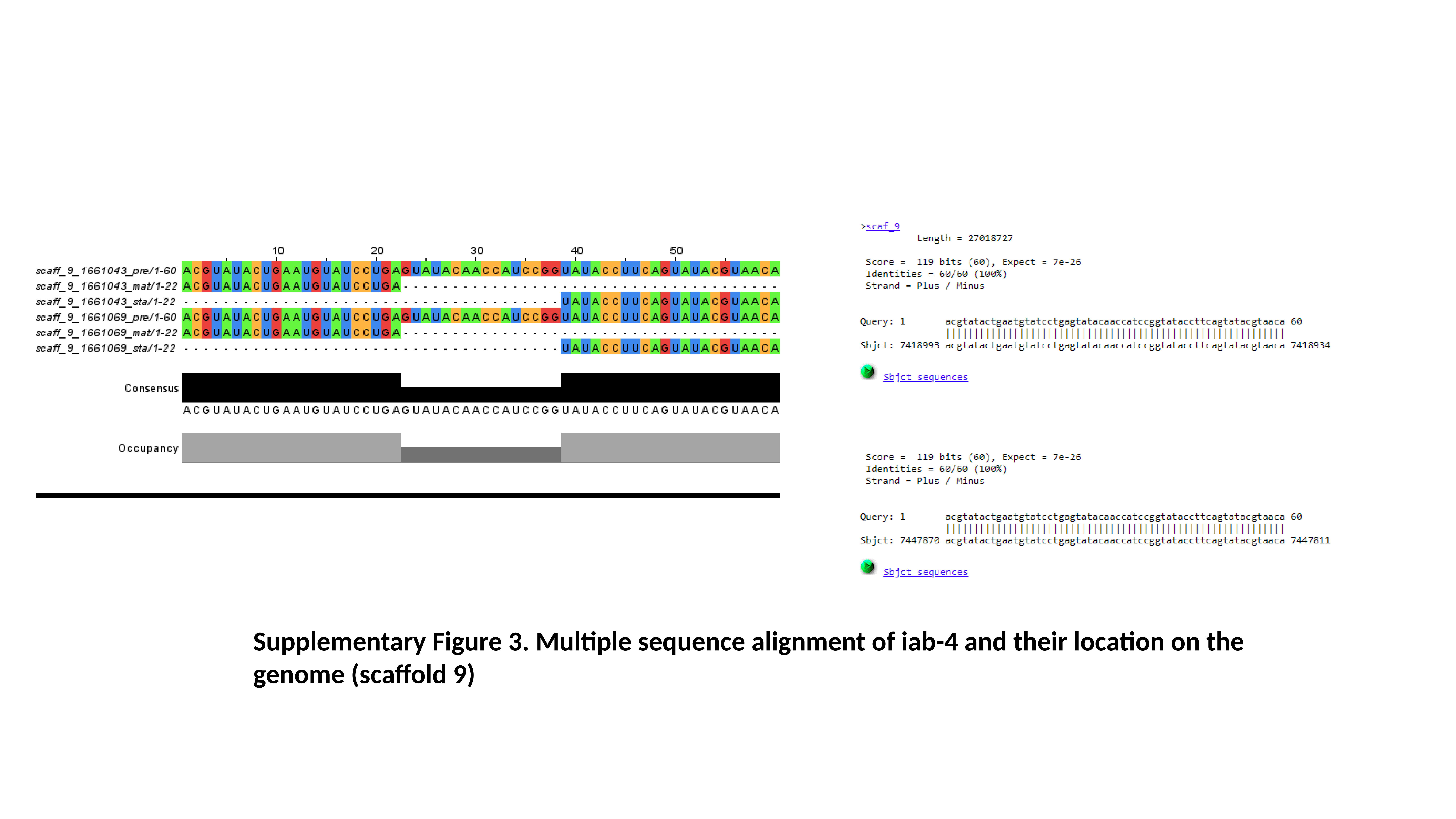

Supplementary Figure 3. Multiple sequence alignment of iab-4 and their location on the genome (scaffold 9)

## Slide 4
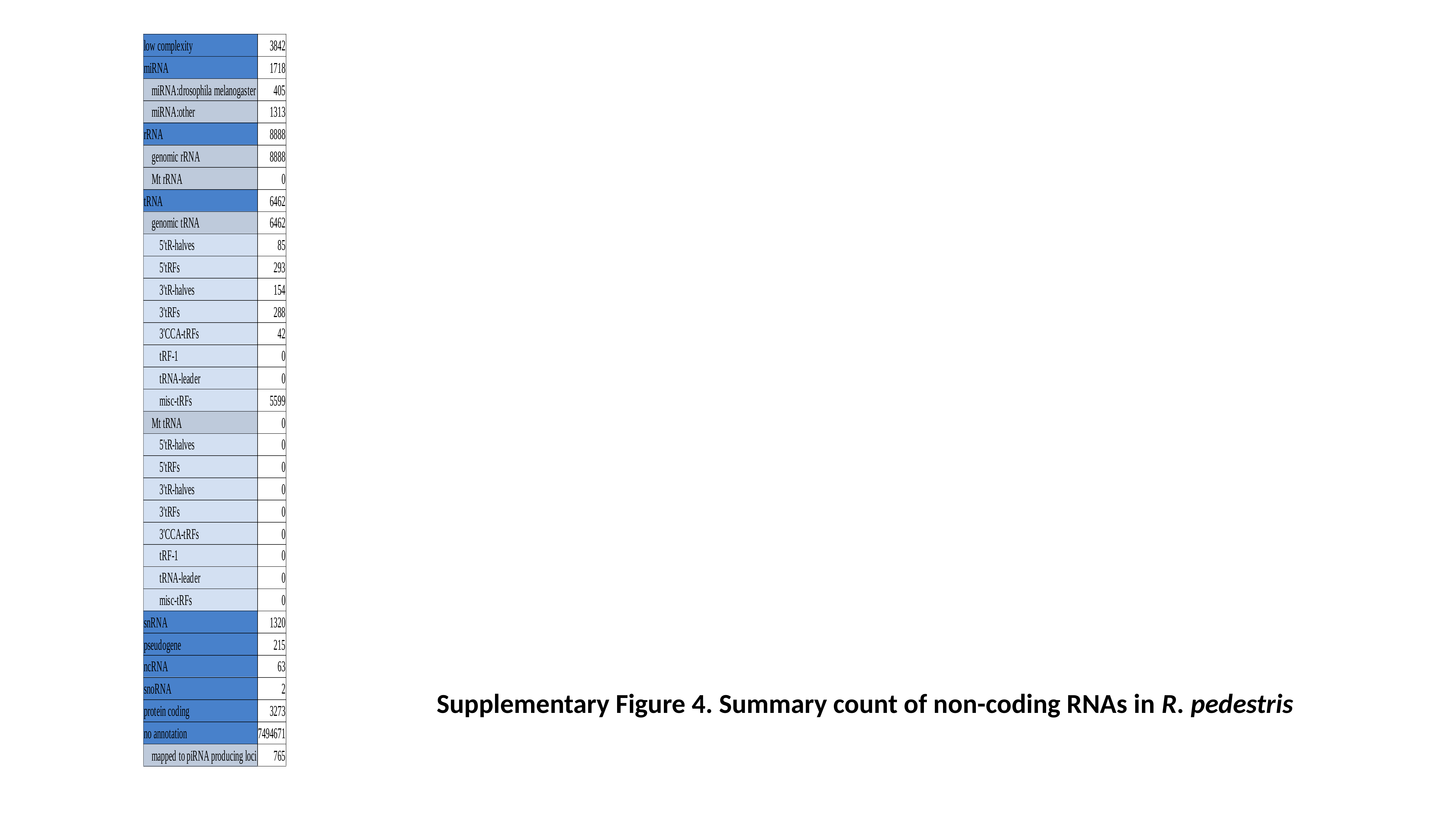

Supplementary Figure 4. Summary count of non-coding RNAs in R. pedestris

## Slide 5
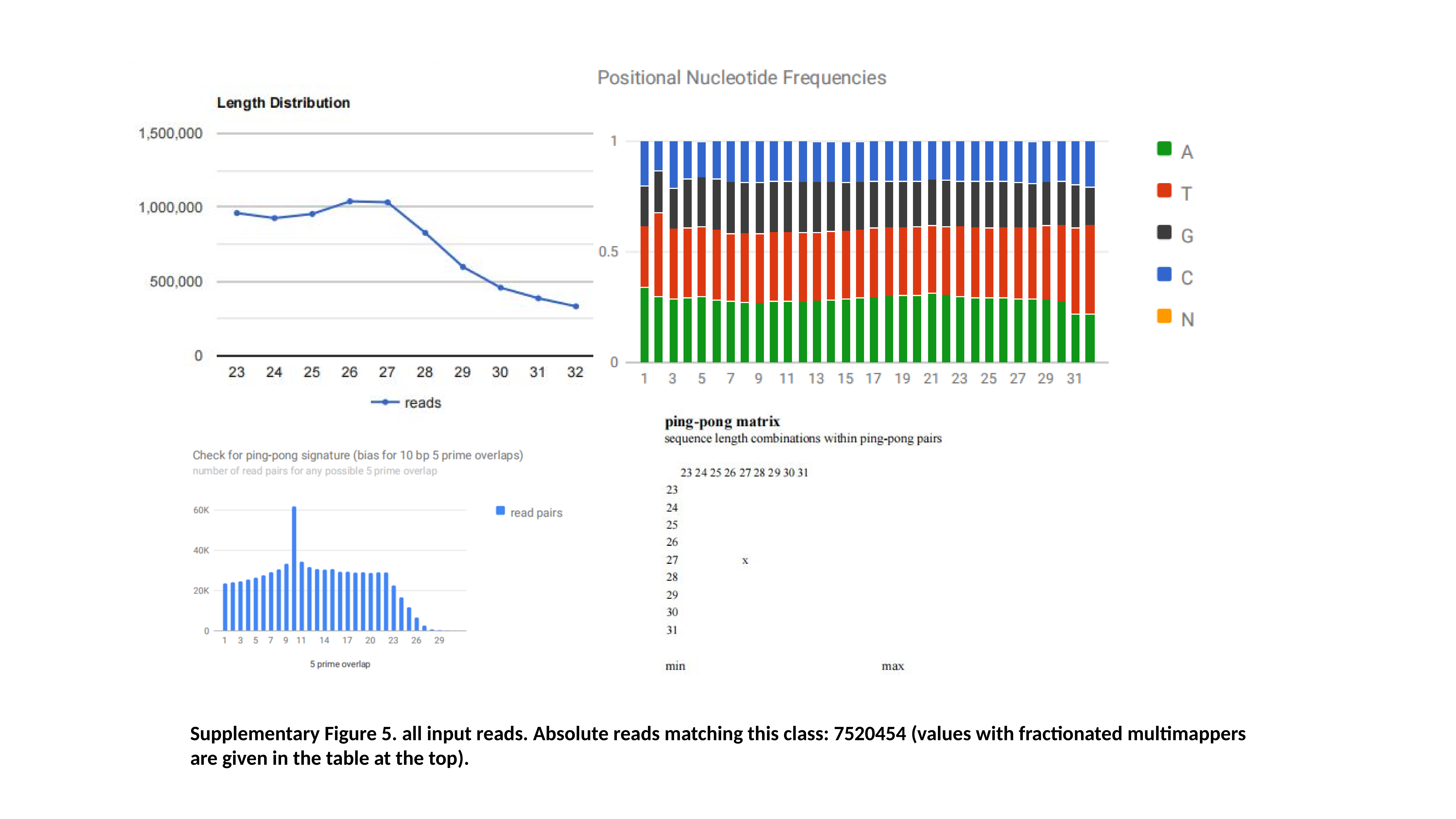

Supplementary Figure 5. all input reads. Absolute reads matching this class: 7520454 (values with fractionated multimappers are given in the table at the top).

## Slide 6
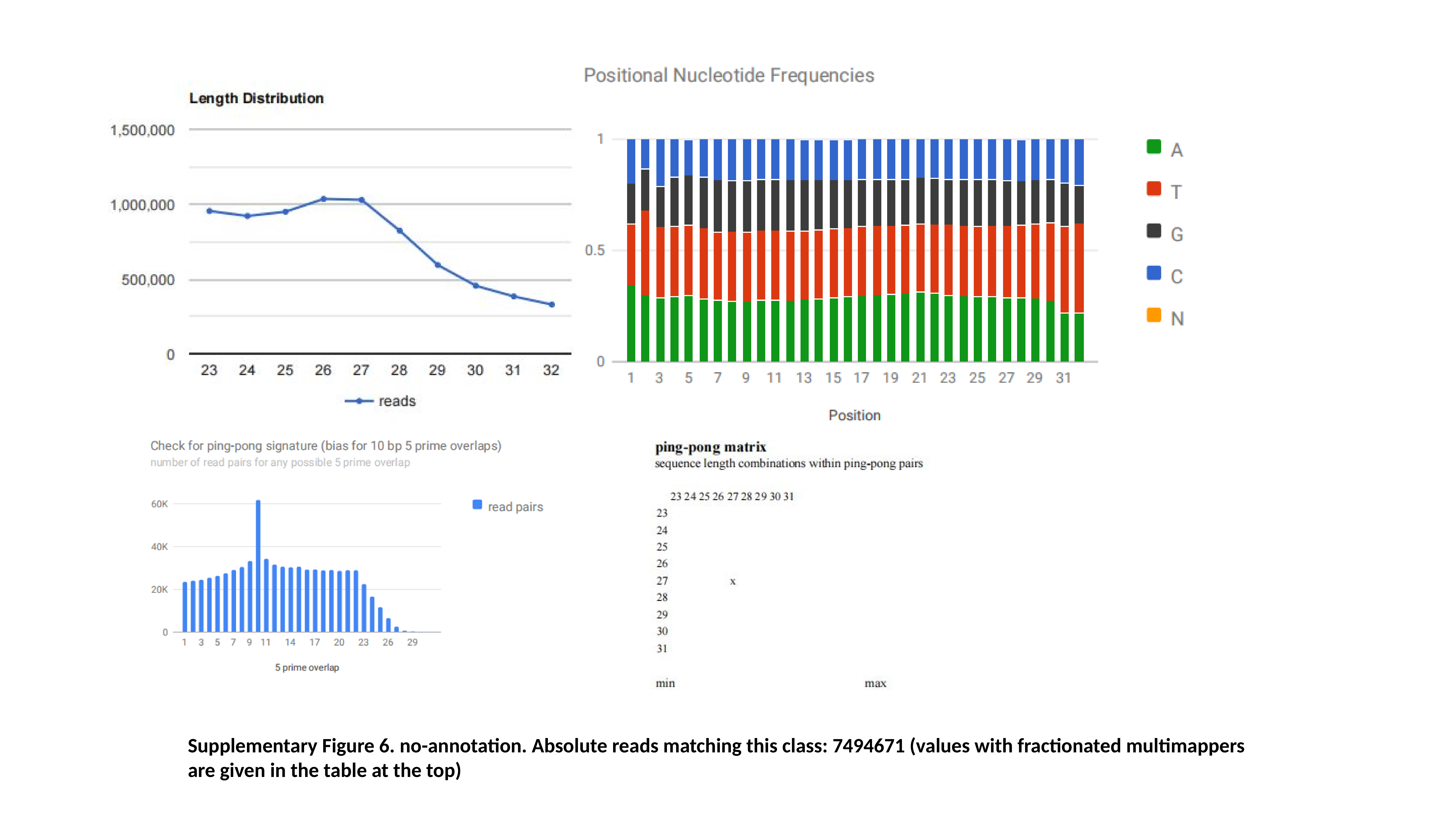

Supplementary Figure 6. no-annotation. Absolute reads matching this class: 7494671 (values with fractionated multimappers are given in the table at the top)

## Slide 7
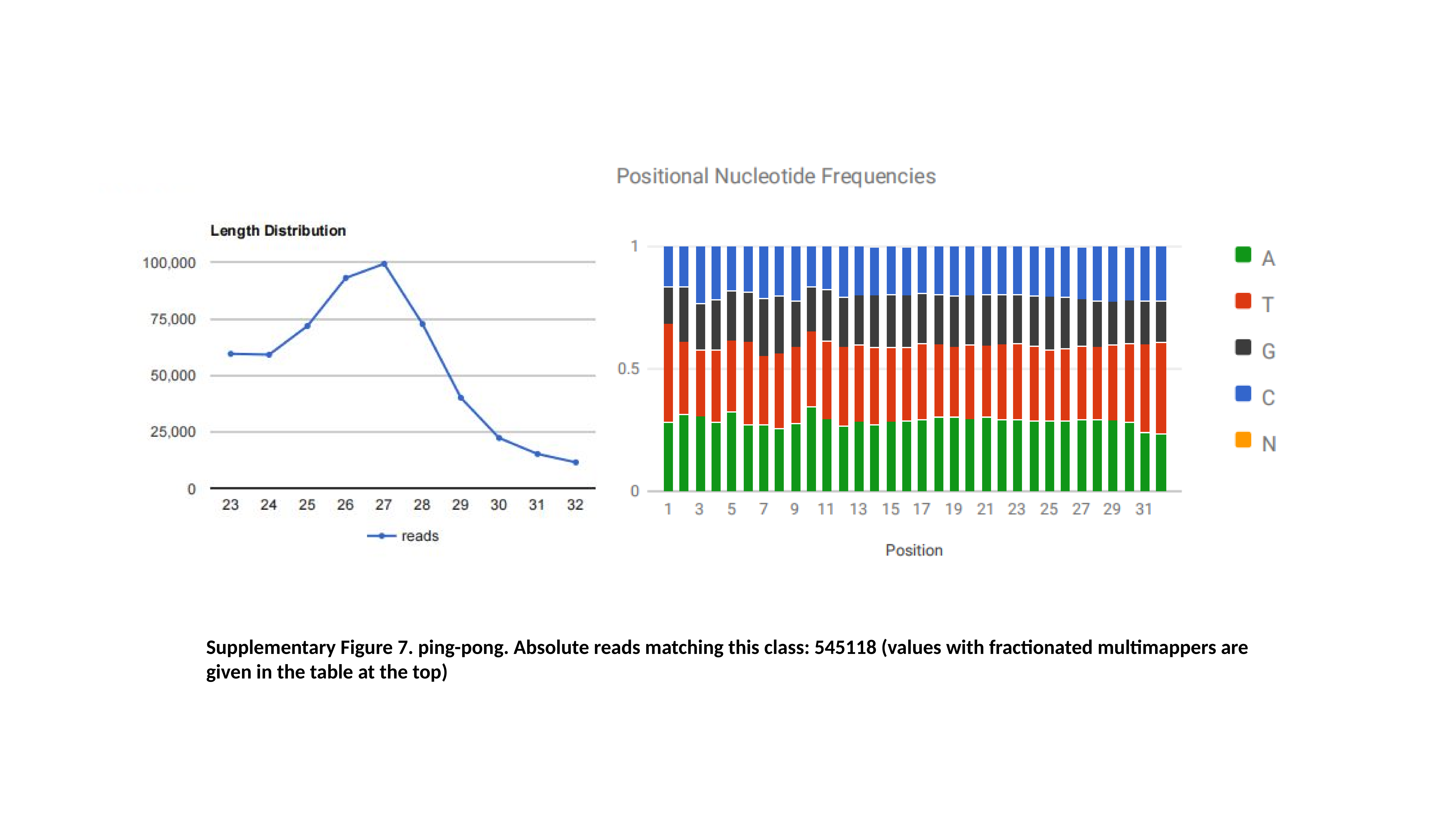

Supplementary Figure 7. ping-pong. Absolute reads matching this class: 545118 (values with fractionated multimappers are given in the table at the top)

## Slide 8
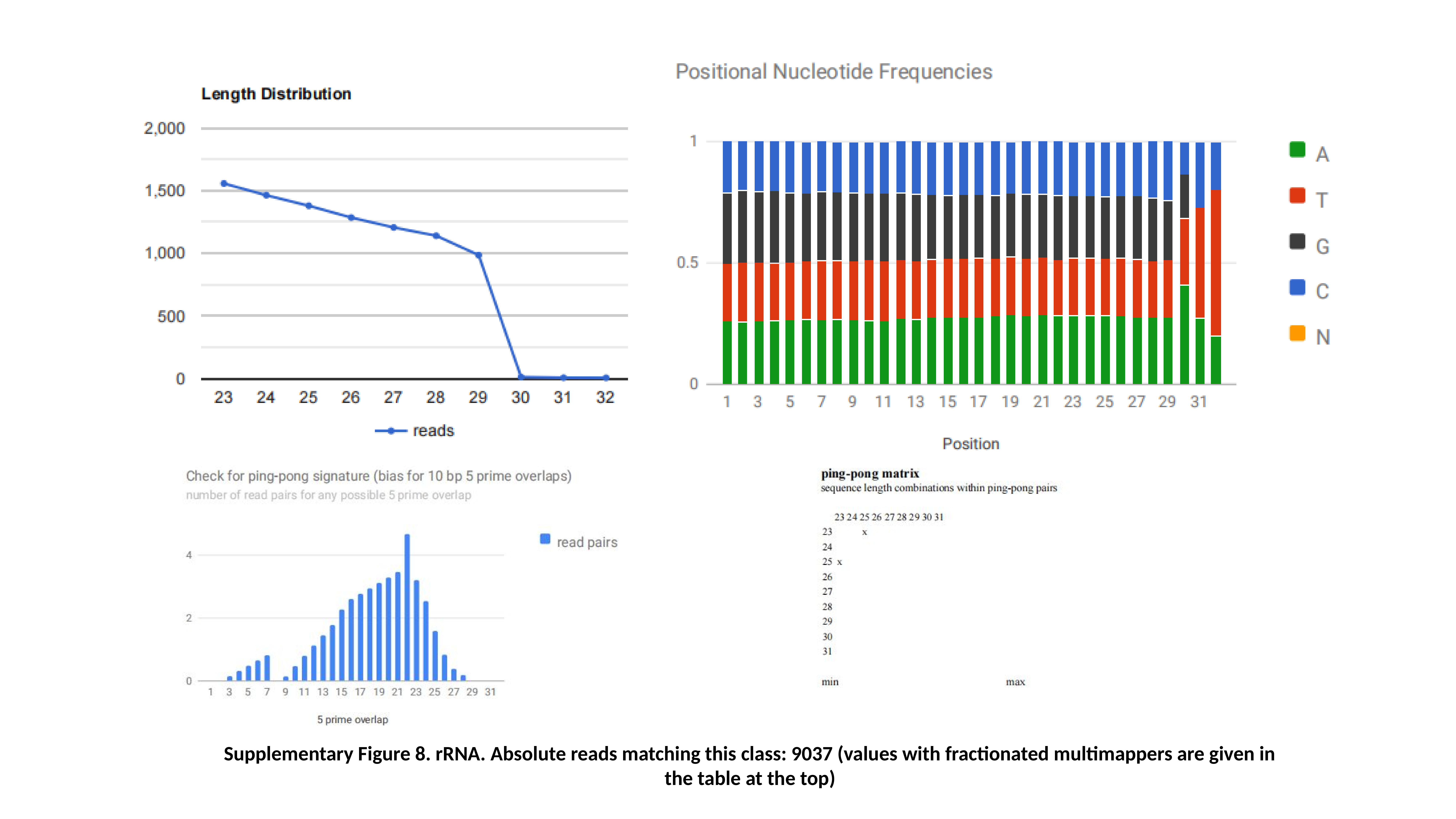

Supplementary Figure 8. rRNA. Absolute reads matching this class: 9037 (values with fractionated multimappers are given in the table at the top)

## Slide 9
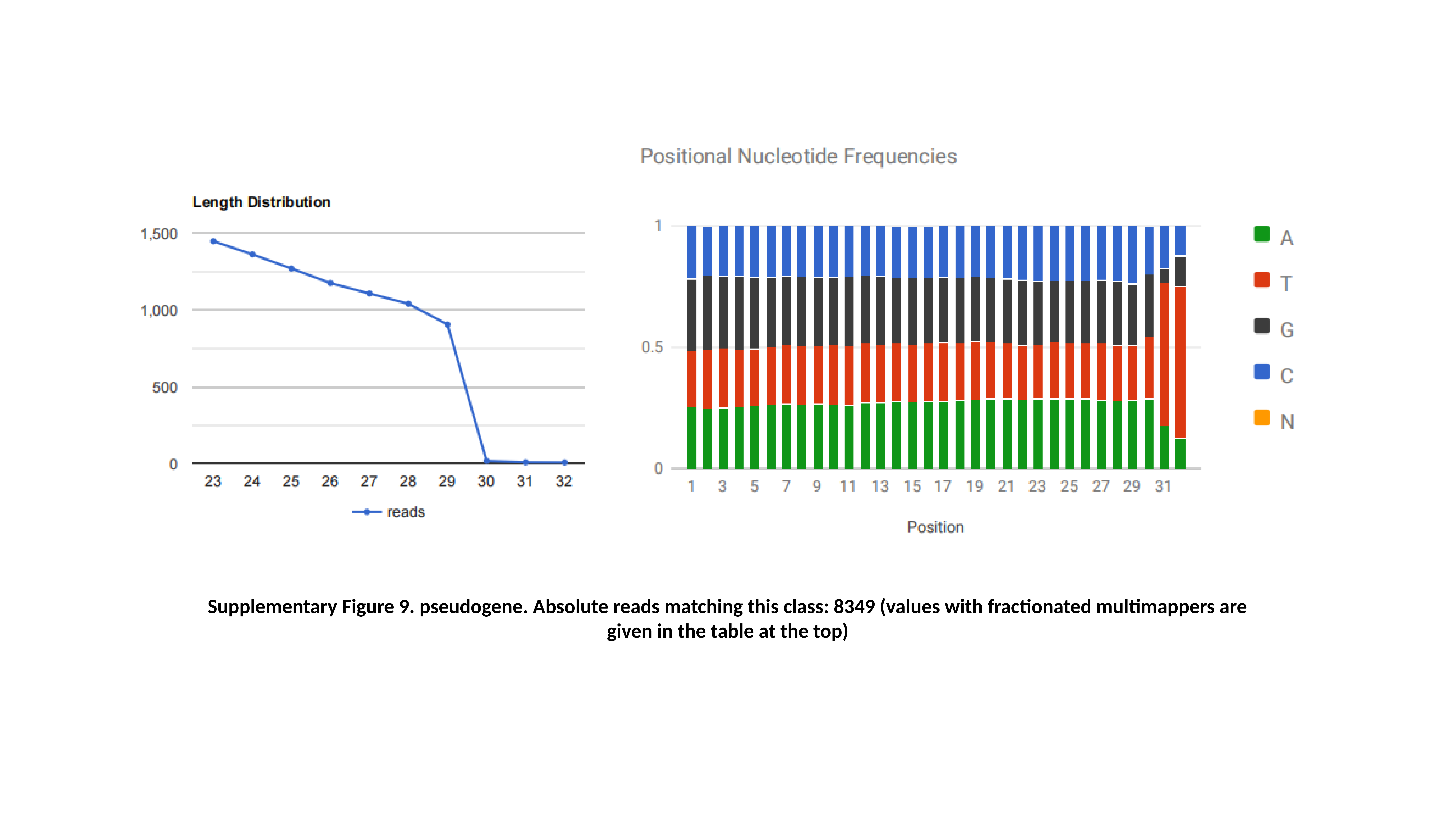

Supplementary Figure 9. pseudogene. Absolute reads matching this class: 8349 (values with fractionated multimappers are given in the table at the top)

## Slide 10
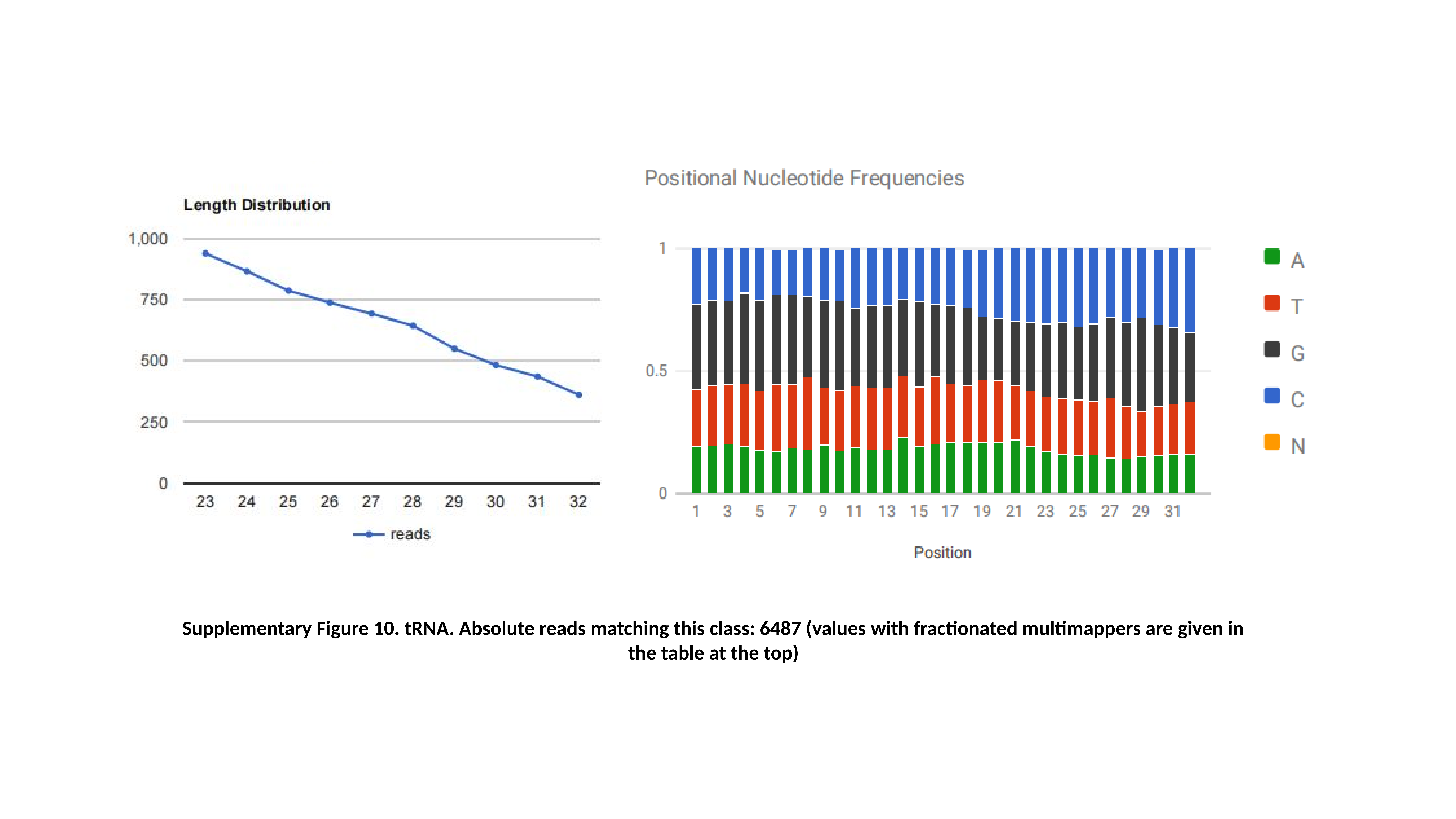

Supplementary Figure 10. tRNA. Absolute reads matching this class: 6487 (values with fractionated multimappers are given in the table at the top)

## Slide 11
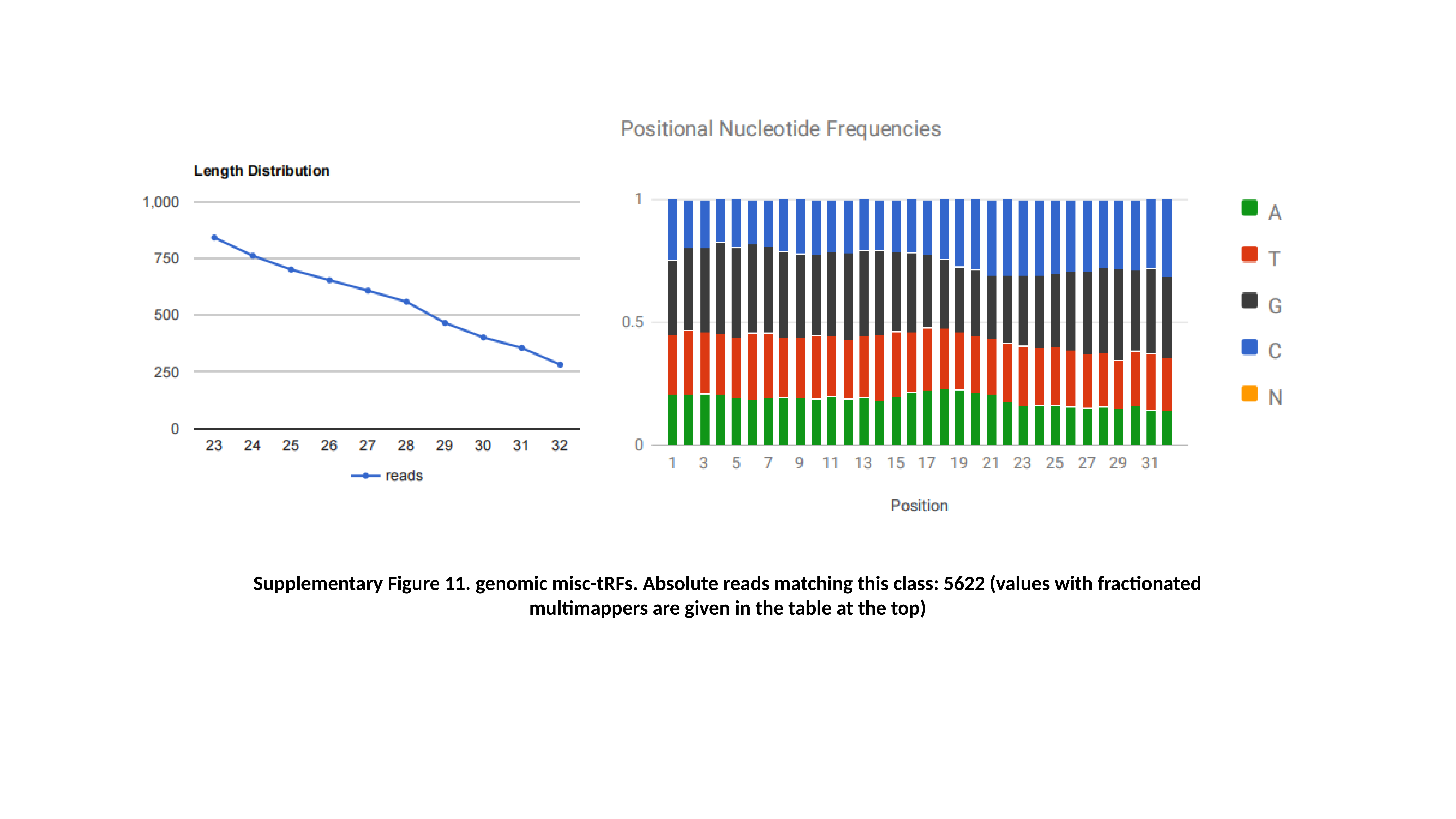

Supplementary Figure 11. genomic misc-tRFs. Absolute reads matching this class: 5622 (values with fractionated multimappers are given in the table at the top)

## Slide 12
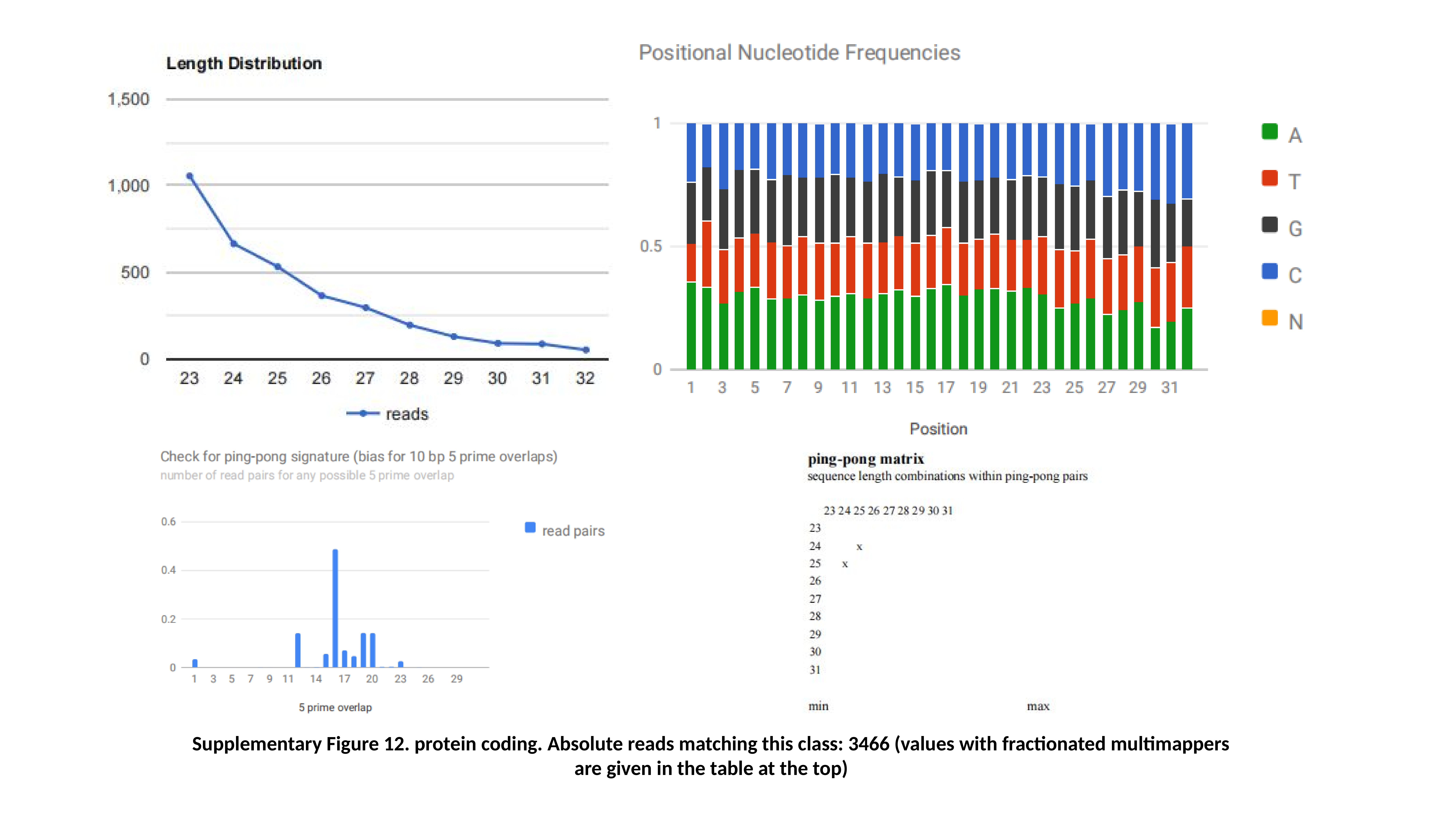

Supplementary Figure 12. protein coding. Absolute reads matching this class: 3466 (values with fractionated multimappers are given in the table at the top)

## Slide 13
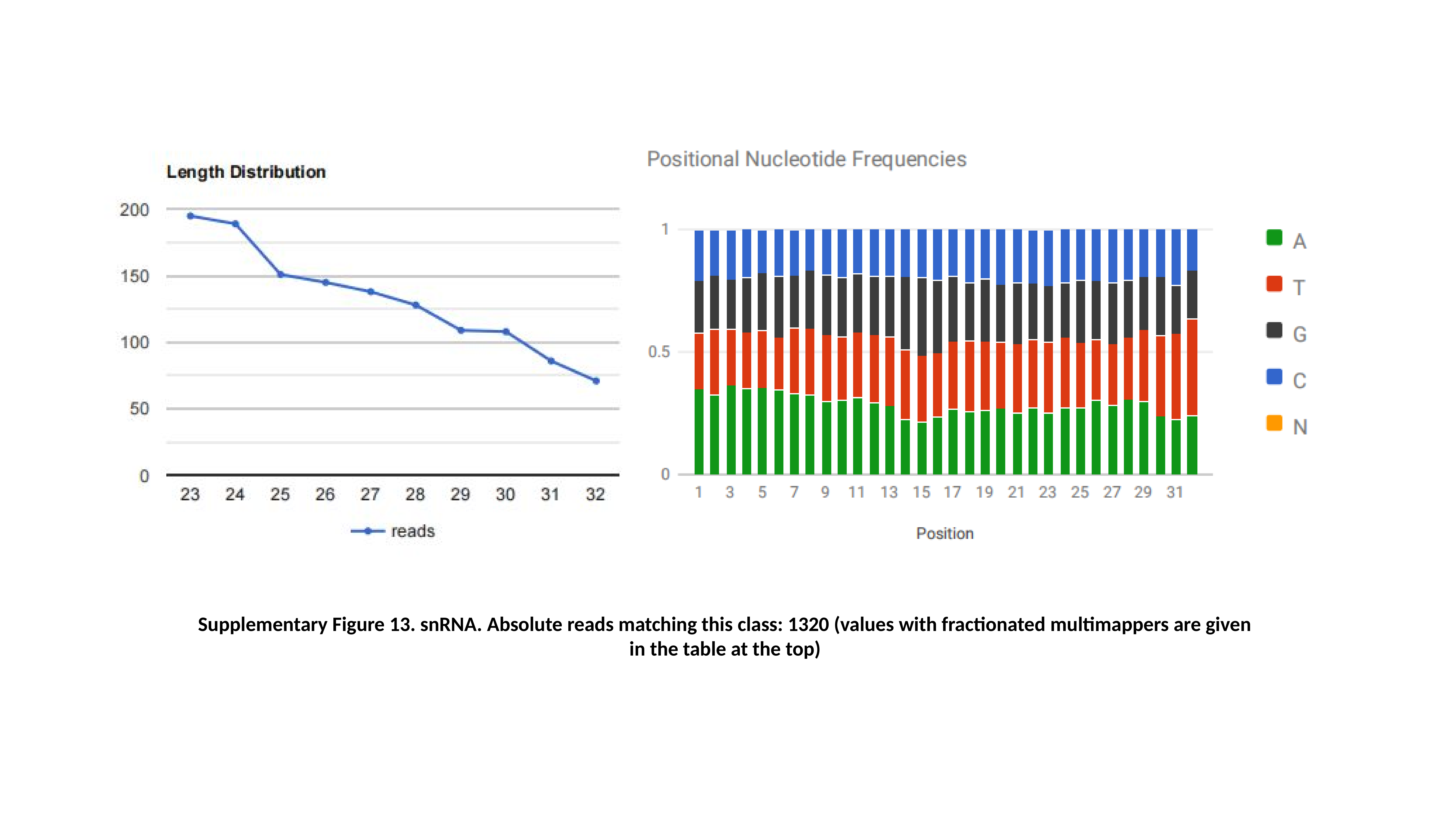

Supplementary Figure 13. snRNA. Absolute reads matching this class: 1320 (values with fractionated multimappers are given in the table at the top)

## Slide 14
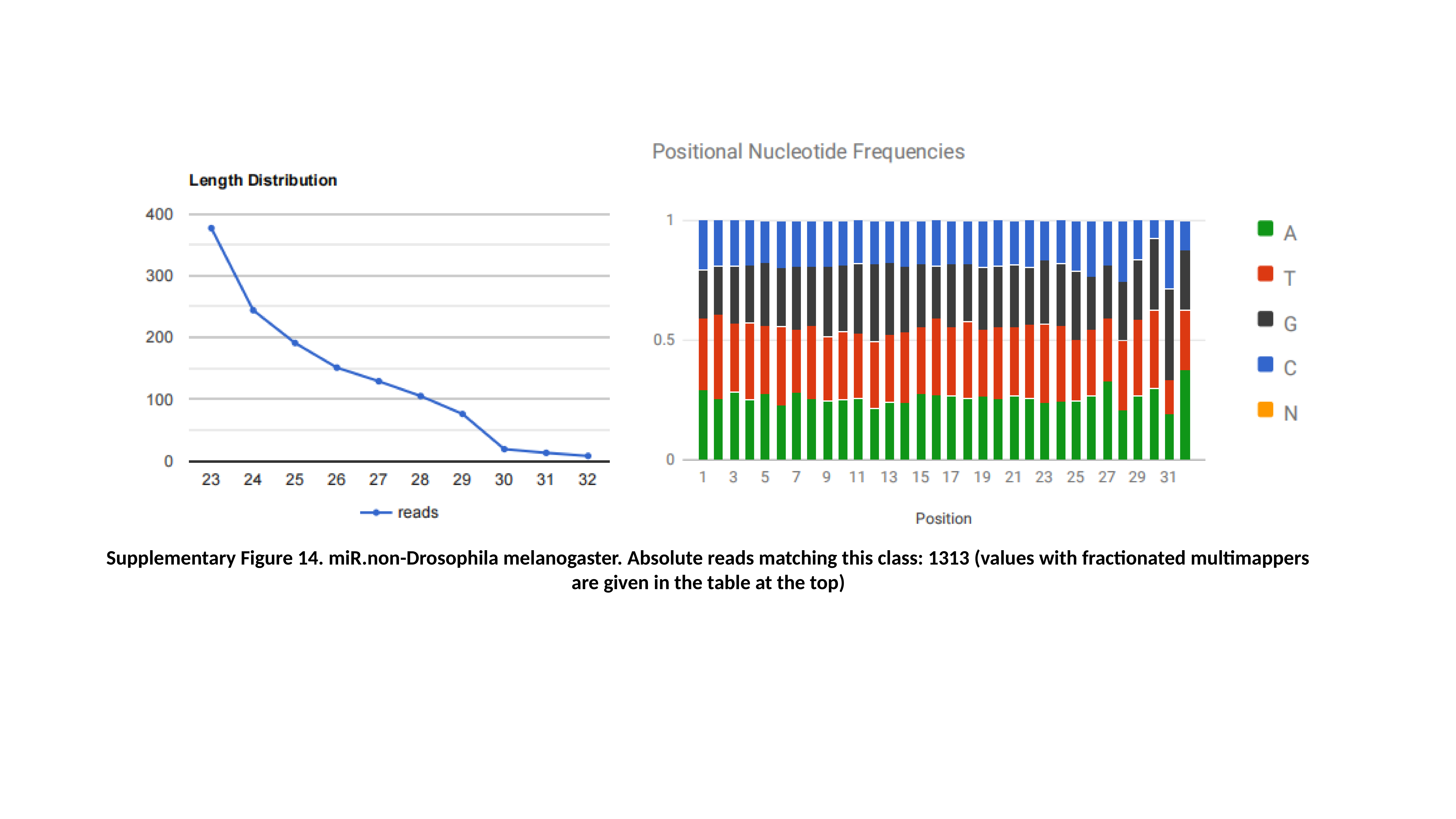

Supplementary Figure 14. miR.non-Drosophila melanogaster. Absolute reads matching this class: 1313 (values with fractionated multimappers are given in the table at the top)

## Slide 15
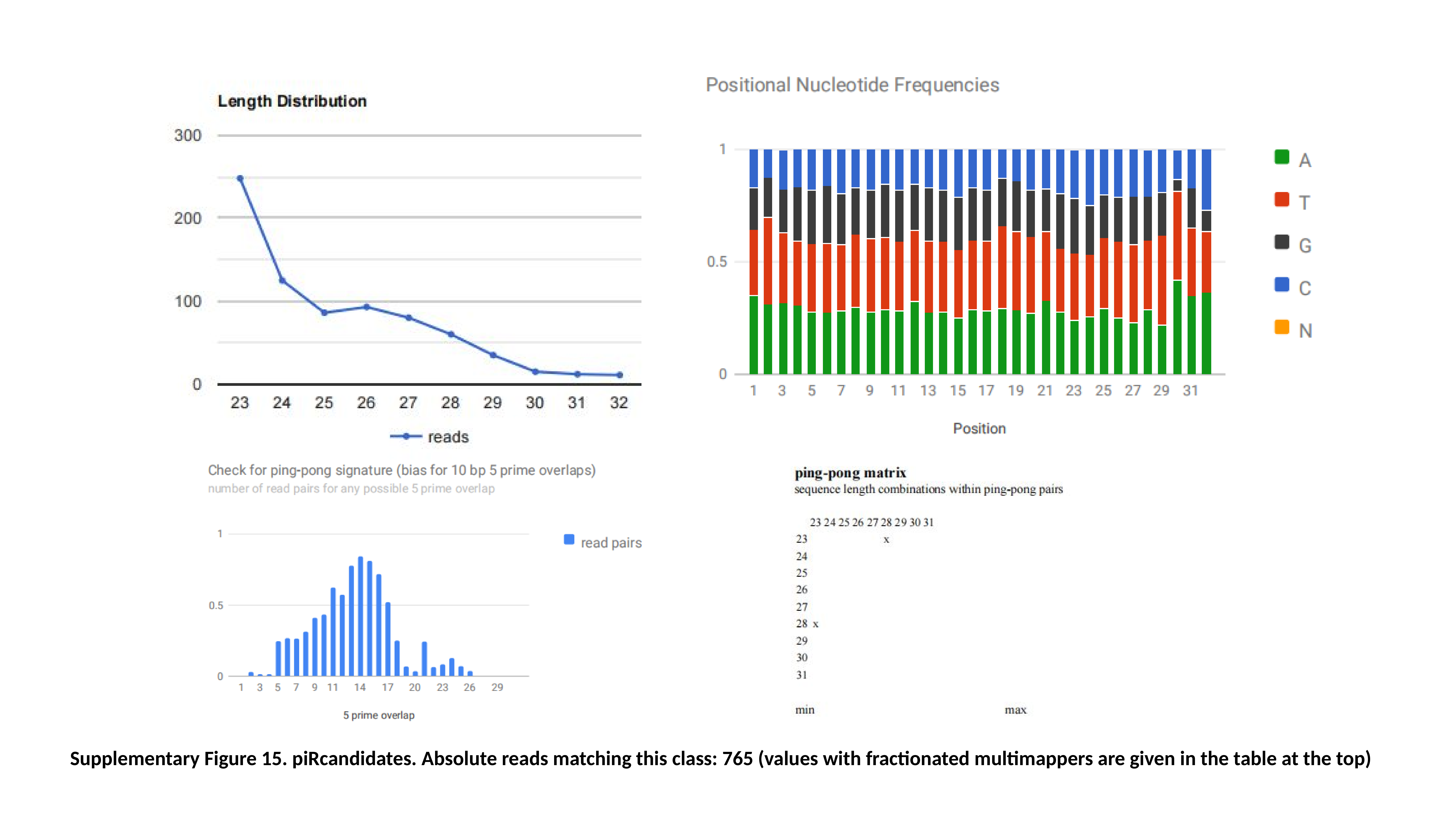

Supplementary Figure 15. piRcandidates. Absolute reads matching this class: 765 (values with fractionated multimappers are given in the table at the top)

## Slide 16
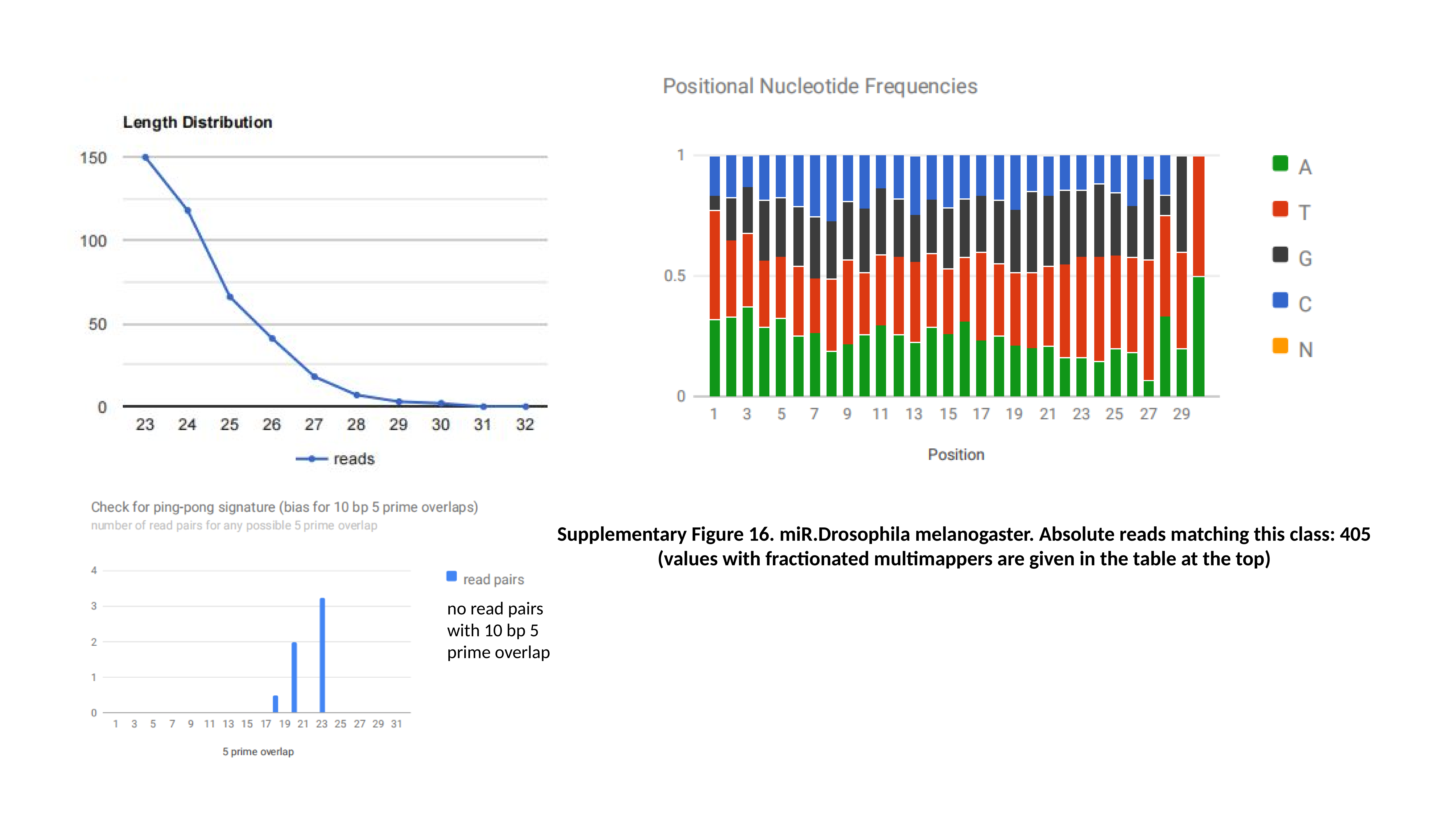

Supplementary Figure 16. miR.Drosophila melanogaster. Absolute reads matching this class: 405 (values with fractionated multimappers are given in the table at the top)
no read pairs with 10 bp 5 prime overlap

## Slide 17
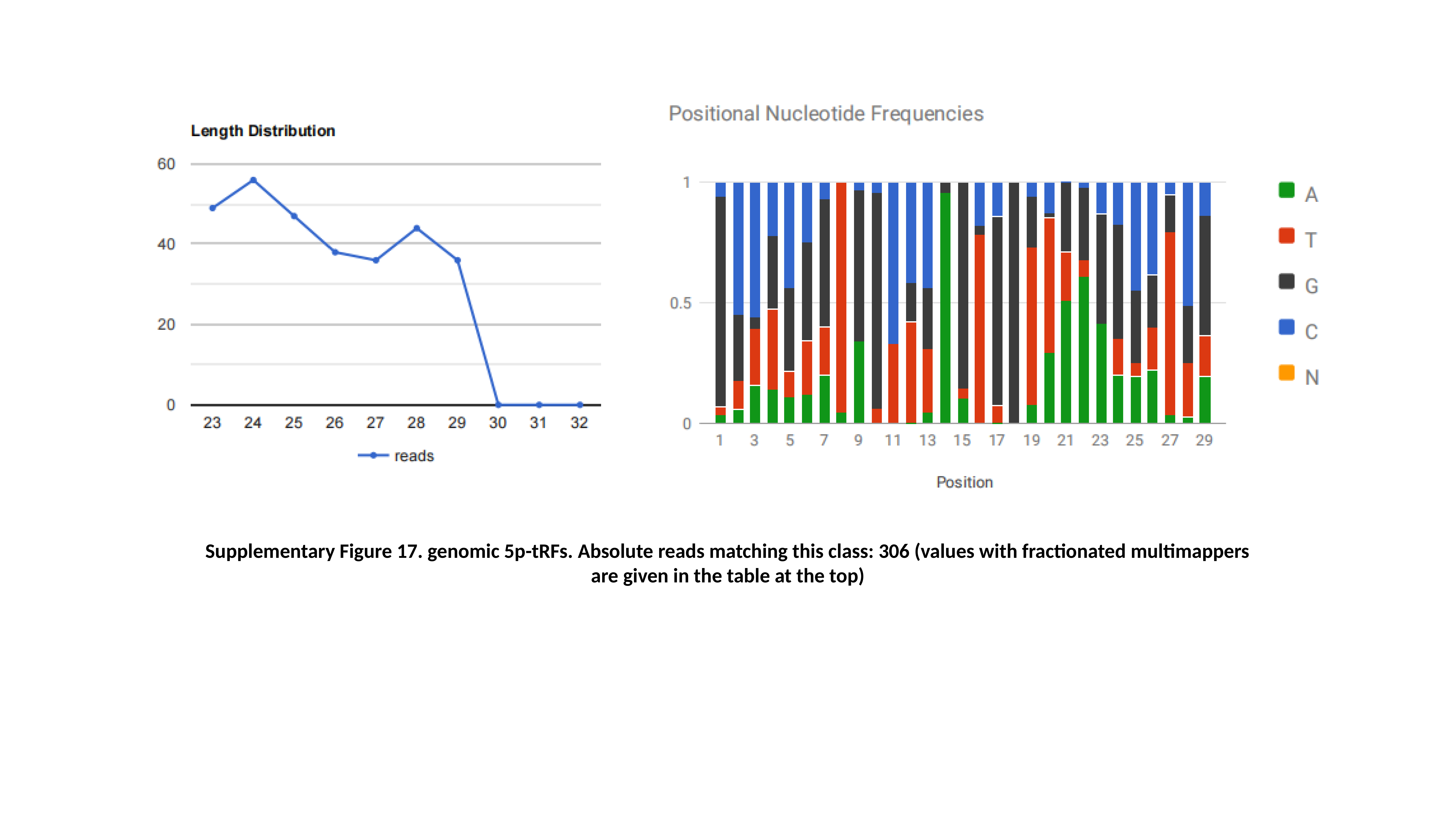

Supplementary Figure 17. genomic 5p-tRFs. Absolute reads matching this class: 306 (values with fractionated multimappers are given in the table at the top)

## Slide 18
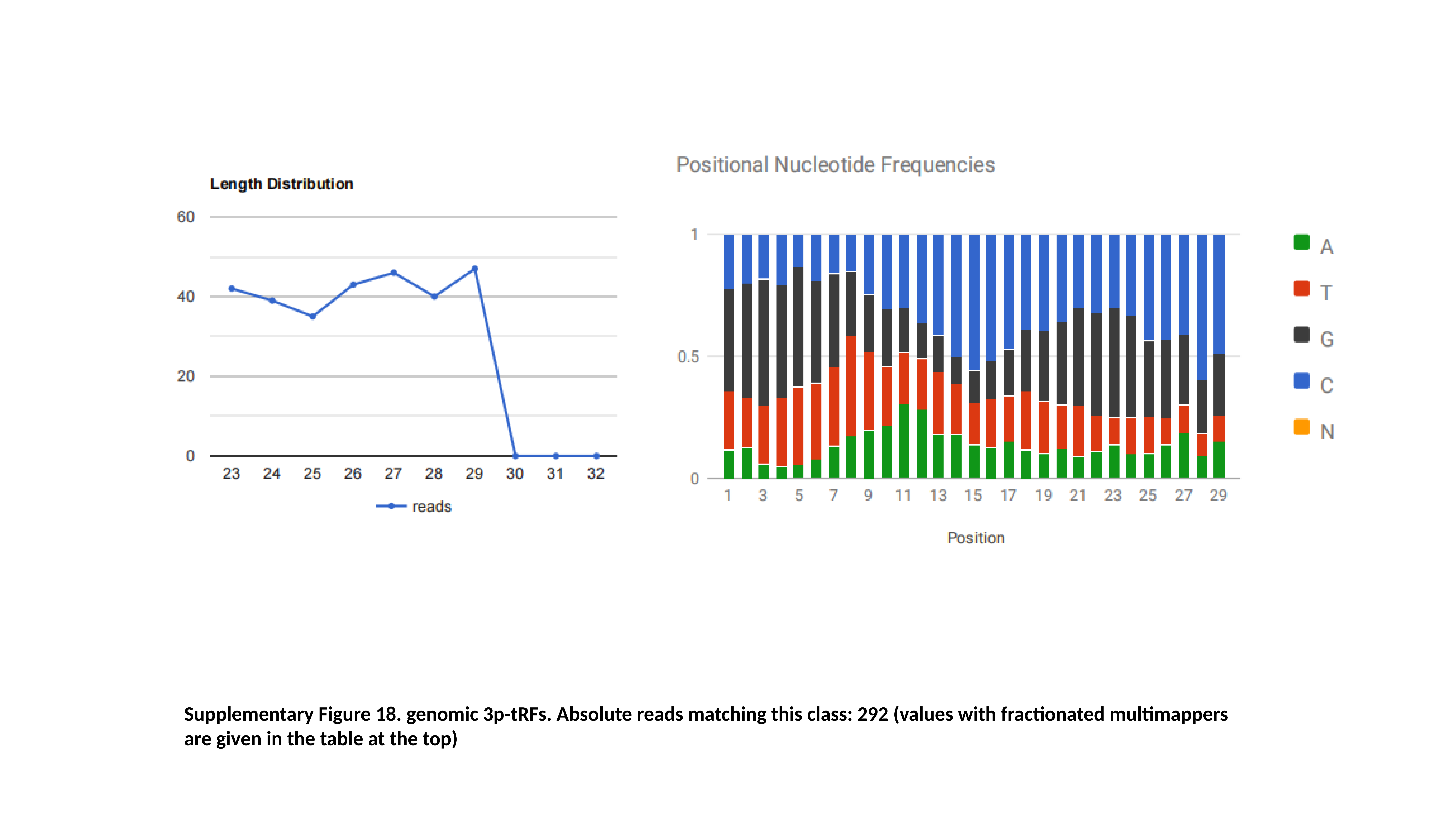

Supplementary Figure 18. genomic 3p-tRFs. Absolute reads matching this class: 292 (values with fractionated multimappers are given in the table at the top)

## Slide 19
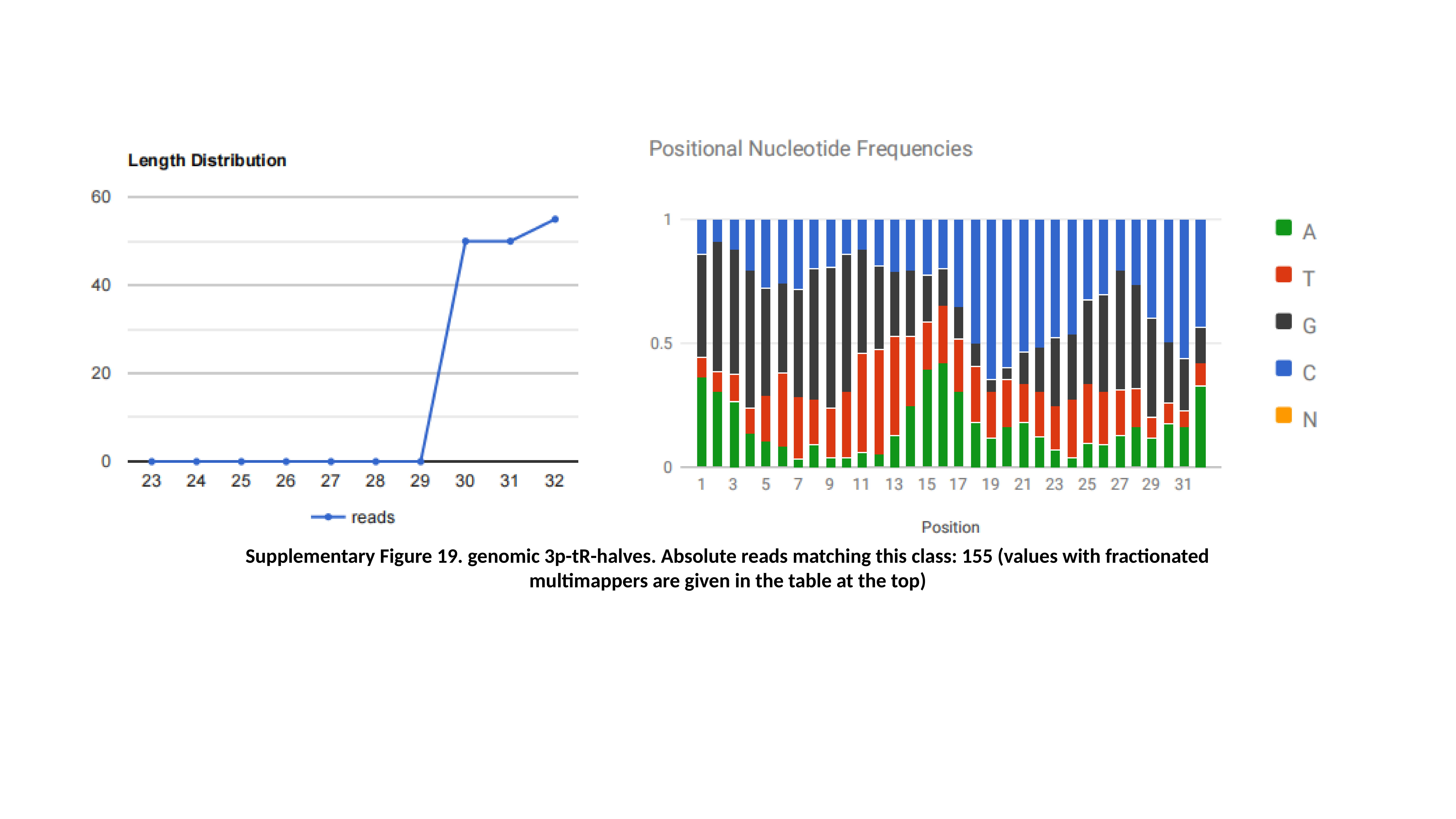

Supplementary Figure 19. genomic 3p-tR-halves. Absolute reads matching this class: 155 (values with fractionated multimappers are given in the table at the top)

## Slide 20
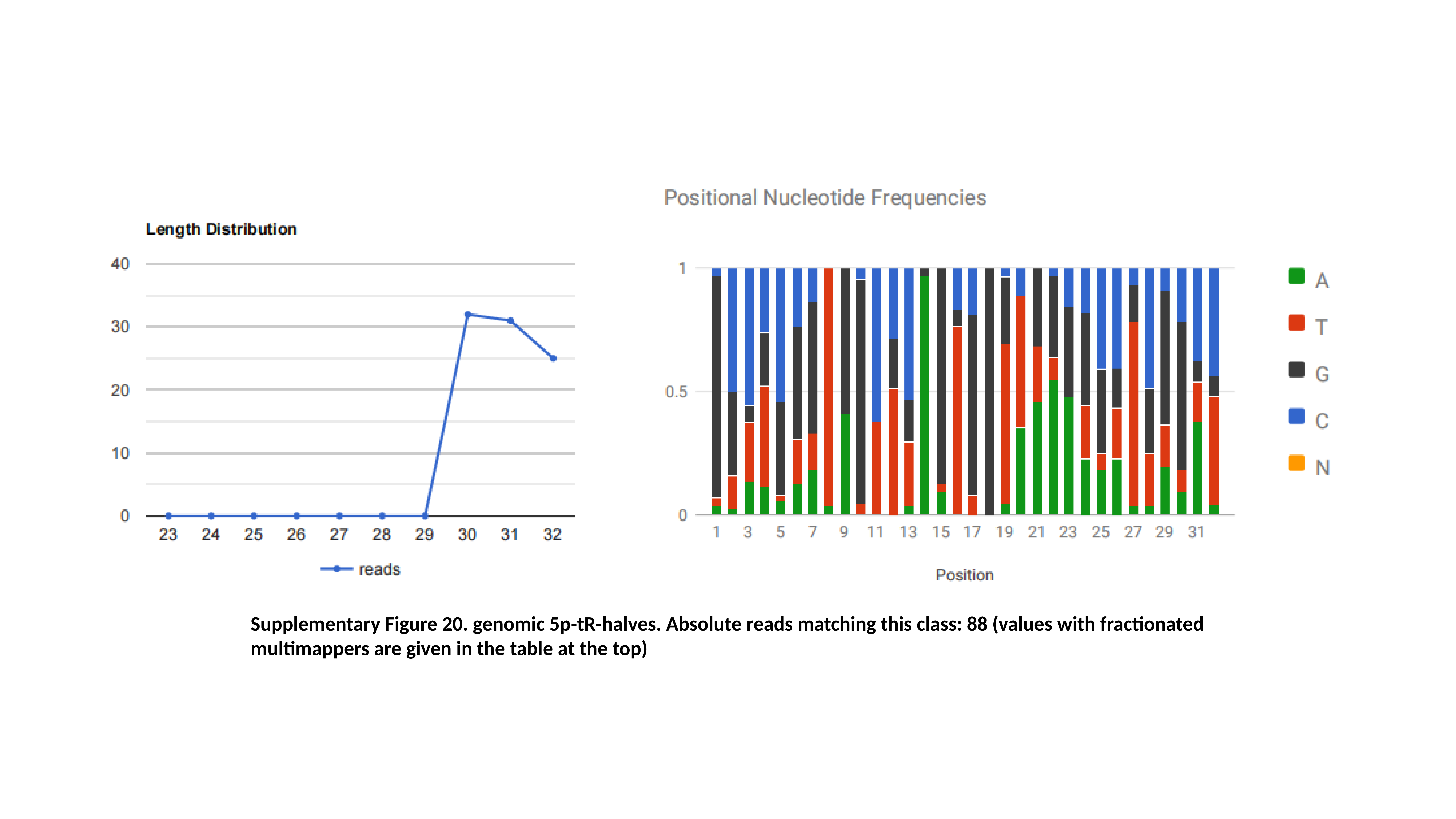

Supplementary Figure 20. genomic 5p-tR-halves. Absolute reads matching this class: 88 (values with fractionated multimappers are given in the table at the top)

## Slide 21
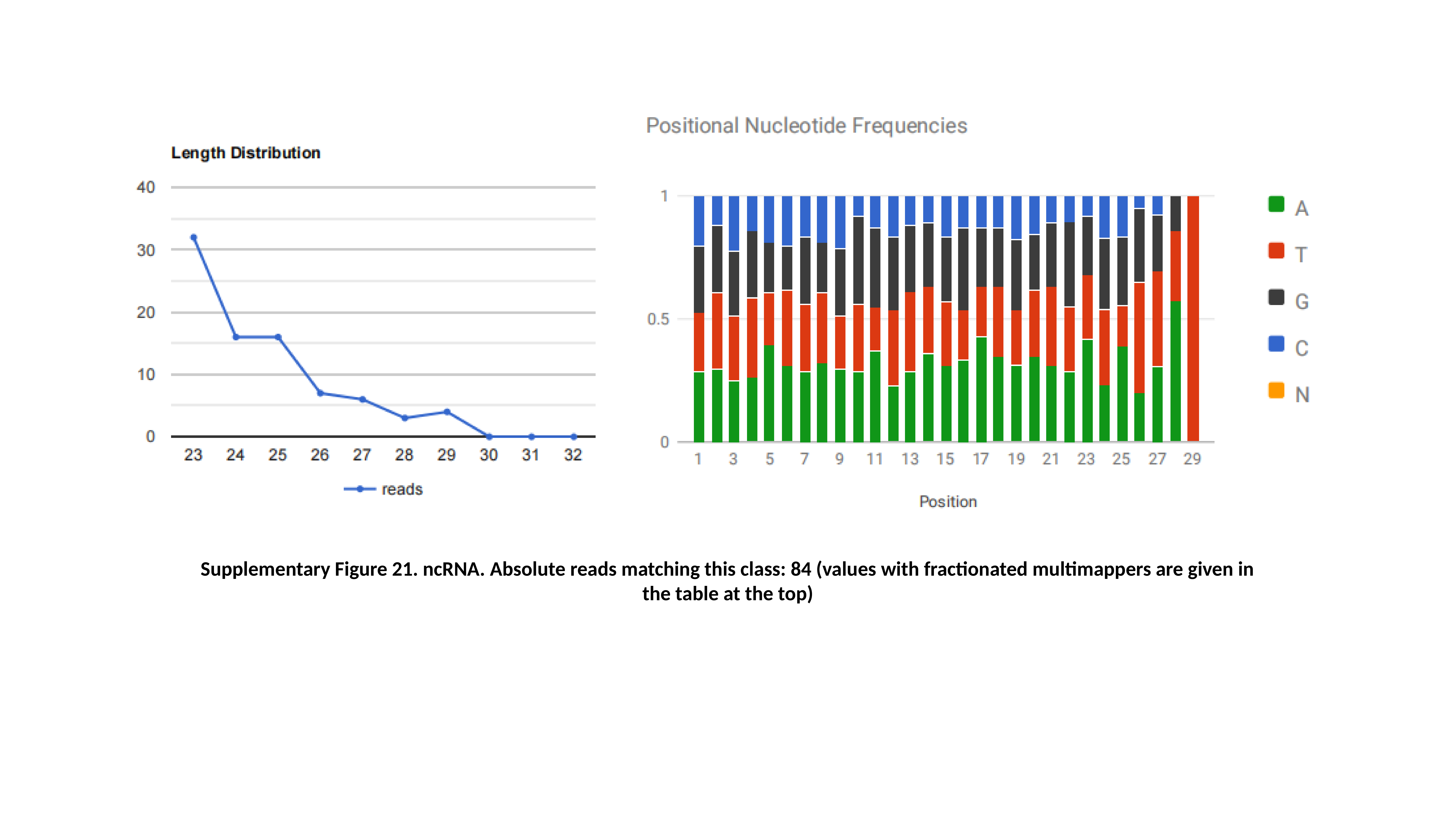

Supplementary Figure 21. ncRNA. Absolute reads matching this class: 84 (values with fractionated multimappers are given in the table at the top)

## Slide 22
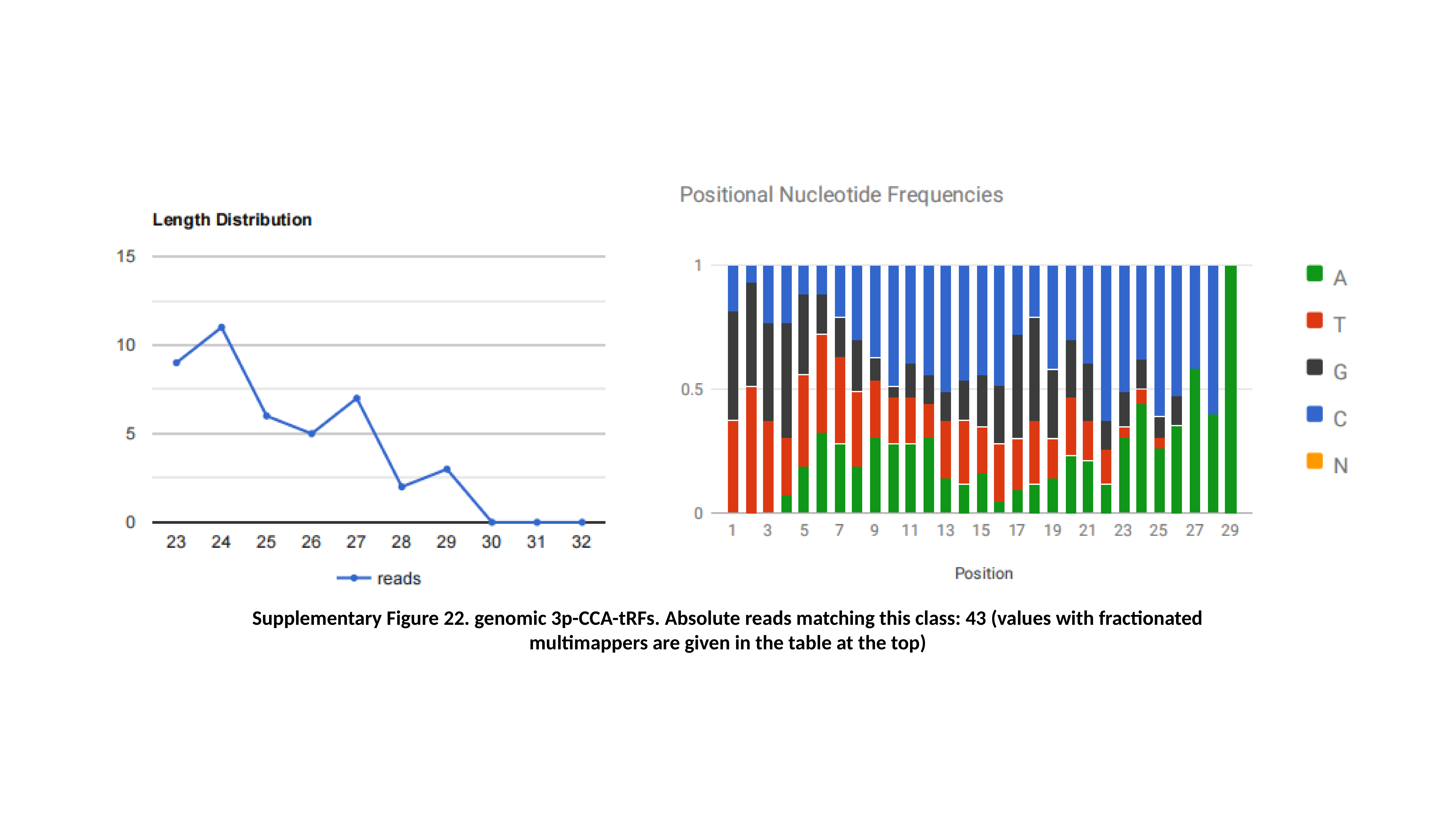

Supplementary Figure 22. genomic 3p-CCA-tRFs. Absolute reads matching this class: 43 (values with fractionated multimappers are given in the table at the top)

## Slide 23
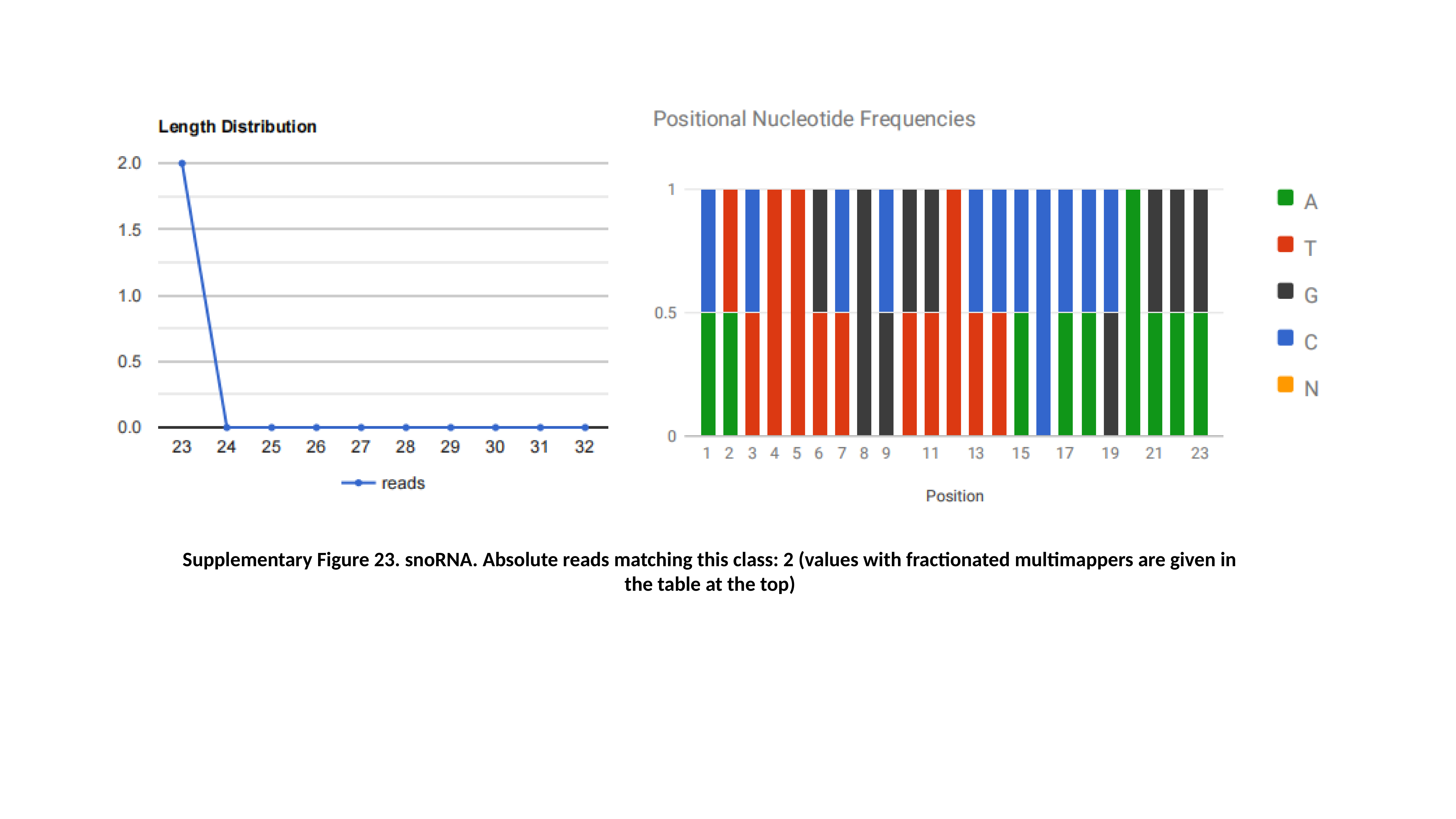

Supplementary Figure 23. snoRNA. Absolute reads matching this class: 2 (values with fractionated multimappers are given in the table at the top)

## Slide 24
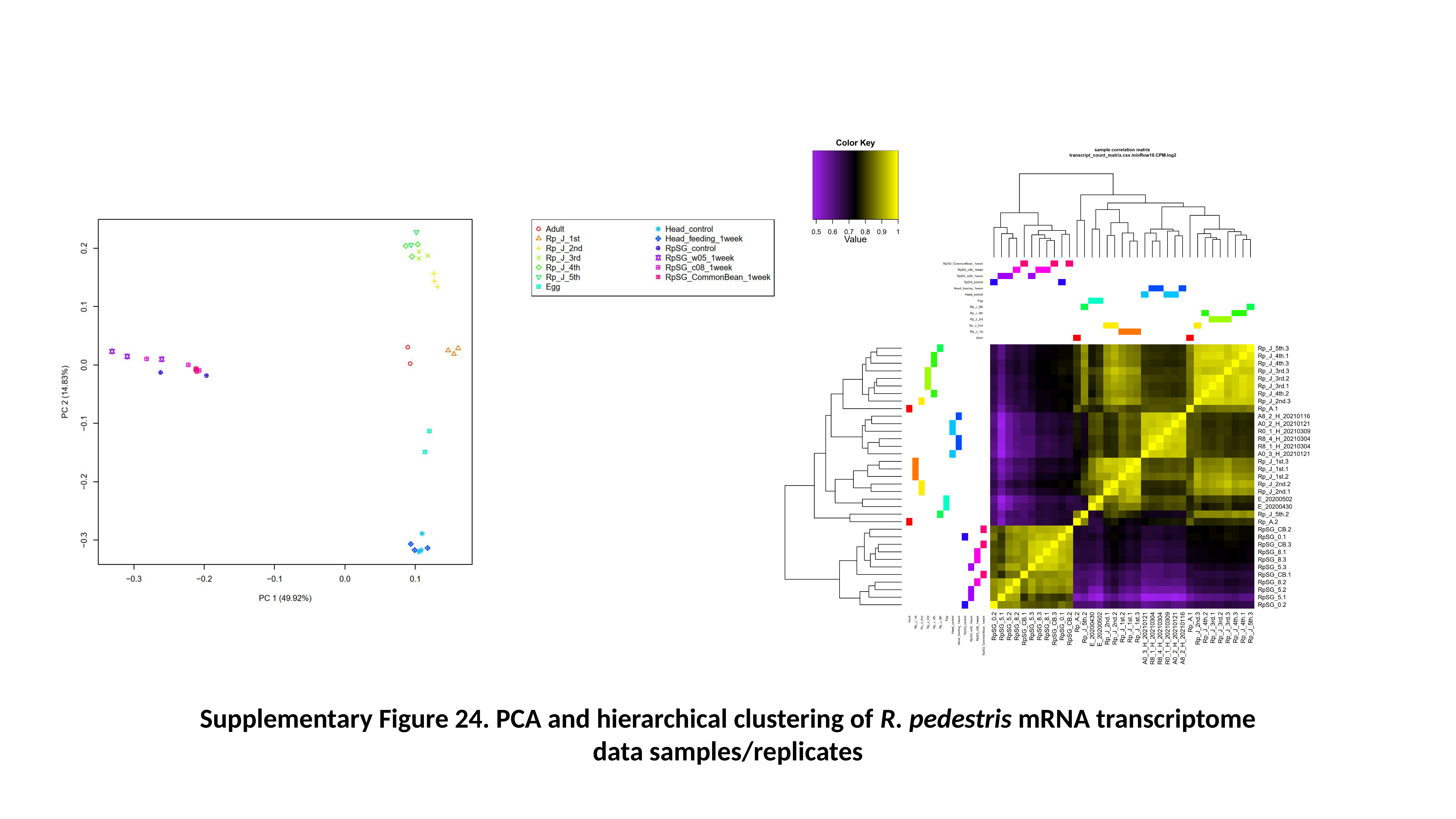

Supplementary Figure 24. PCA and hierarchical clustering of R. pedestris mRNA transcriptome data samples/replicates

## Slide 25
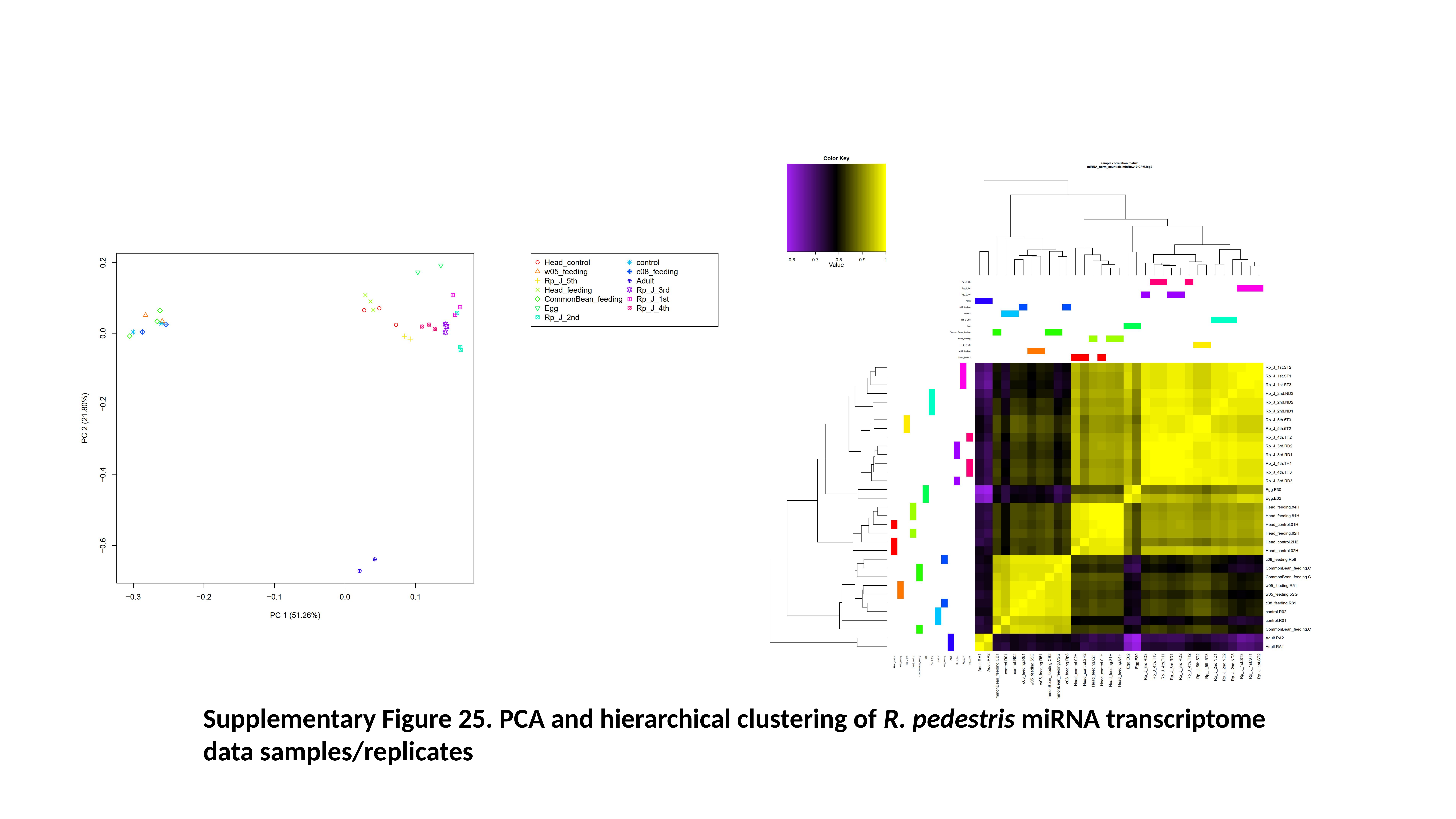

Supplementary Figure 25. PCA and hierarchical clustering of R. pedestris miRNA transcriptome data samples/replicates

## Slide 26
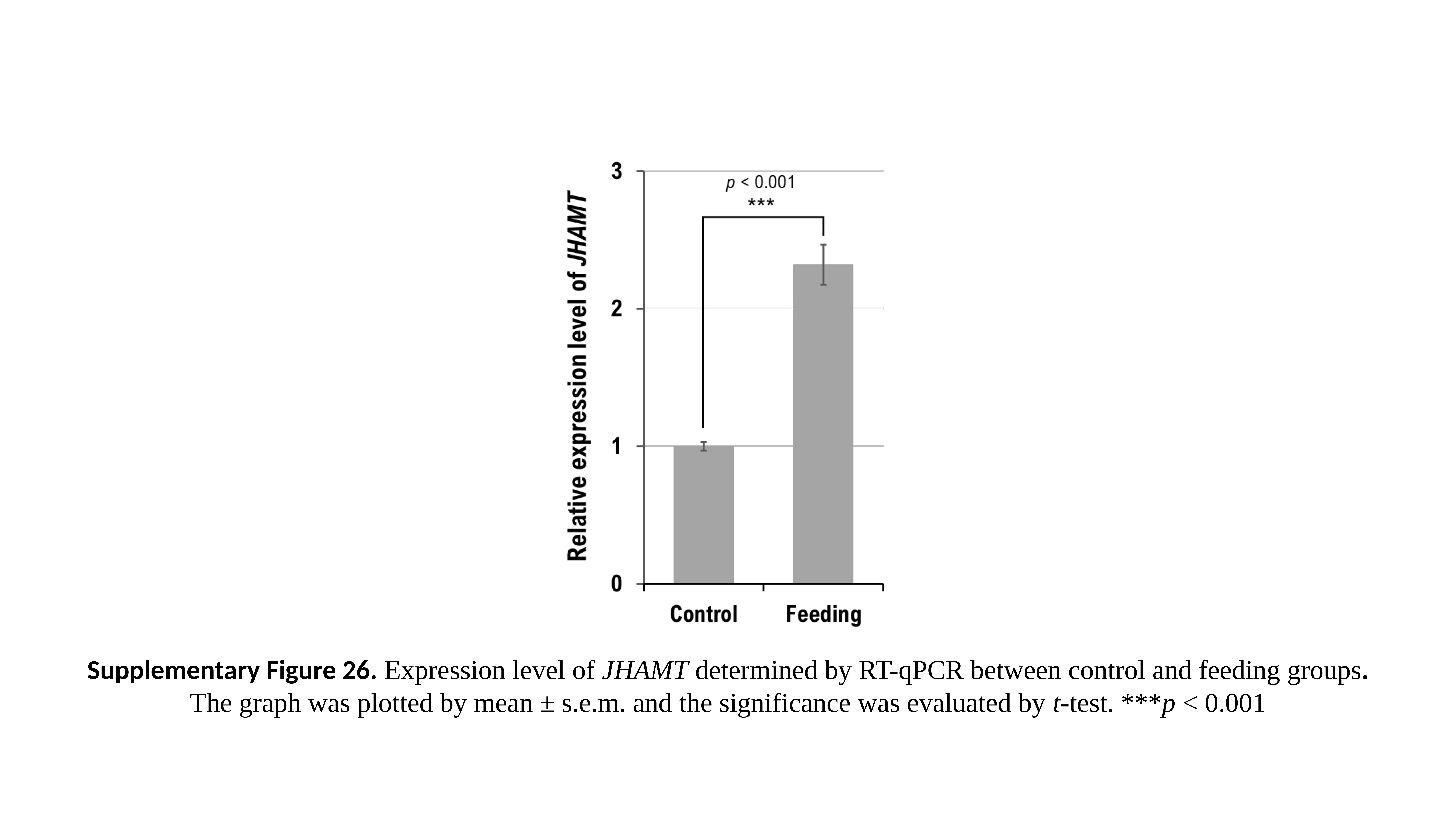

Supplementary Figure 26. Expression level of JHAMT determined by RT-qPCR between control and feeding groups. The graph was plotted by mean ± s.e.m. and the significance was evaluated by t-test. ***p < 0.001

## Slide 27
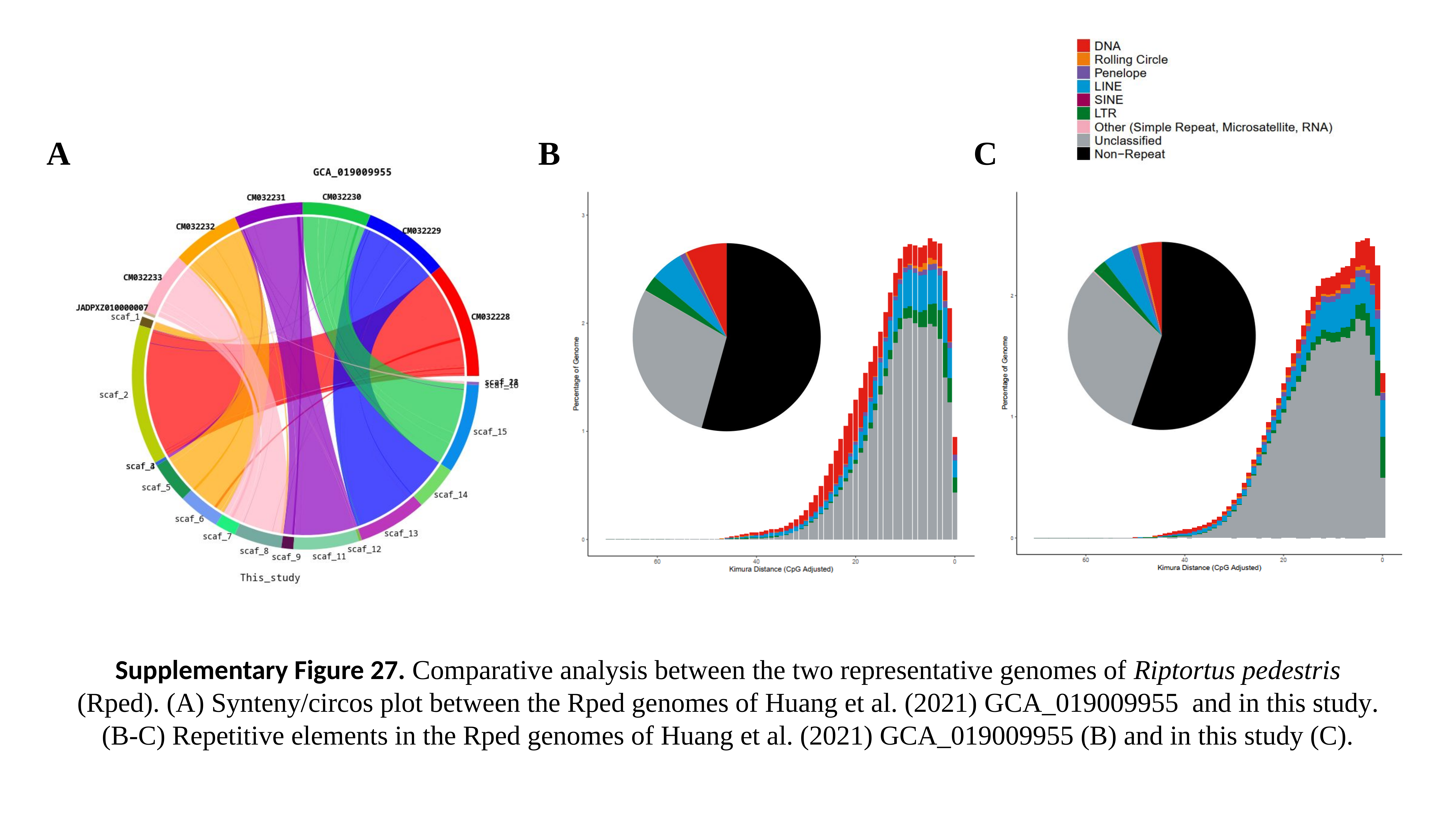

A
B
C
Supplementary Figure 27. Comparative analysis between the two representative genomes of Riptortus pedestris (Rped). (A) Synteny/circos plot between the Rped genomes of Huang et al. (2021) GCA_019009955 and in this study. (B-C) Repetitive elements in the Rped genomes of Huang et al. (2021) GCA_019009955 (B) and in this study (C).
